# Transcriptome Analysis of *Brassica rapa* Near-Isogenic Lines Carrying Clubroot-Resistant and –Susceptible Alleles in Response to *Plasmodiophora brassicae* during Early Infection

**DOI:** 10.3389/fpls.2015.01183

**Published:** 2016-01-05

**Authors:** Jingjing Chen, Wenxing Pang, Bing Chen, Chunyu Zhang, Zhongyun Piao

**Affiliations:** ^1^Department of Horticulture, Shenyang Agricultural UniversityShenyang, China; ^2^Department of Characteristic Horticulture, Taizhou Institute of Agricultural Sciences, Jiangsu Academy of Agricultural SciencesTaizhou, China; ^3^National Key Laboratory of Crop Genetic Improvement and College of Plant Science and Technology, Huazhong Agricultural UniversityWuhan, China

**Keywords:** clubroot, *Plasmodiophora brassicae*, disease resistance, RNA-seq, *Brassica rapa*

## Abstract

Although *Plasmodiophora brassicae* is one of the most common pathogens worldwide, the causal agent of clubroot disease in *Brassica* crops, resistance mechanisms to it are still only poorly understood. To study the early defense response induced by *P. brassicae* infection, a global transcriptome profiling of the roots of two near-isogenic lines (NILs) of clubroot-resistant (CR BJN3-2) and clubroot-susceptible (BJN3-2) Chinese cabbage (*Brassica rapa*) was performed by RNA-seq. Among the 42,730 unique genes mapped to the reference genome of *B. rapa*, 1875, and 2103 genes were found to be up- and down-regulated between CR BJN3-2 and BJN3-2, respectively, at 0, 12, 72, and 96 h after inoculation (hai). Functional annotation showed that most of the differently expressed genes are involved in metabolism, transport, signal transduction, and defense. Of the genes assigned to plant-pathogen interactions, 151 showed different expression patterns between two NILs, including genes associated with pathogen-associated molecular patterns (PAMPs) and effectors recognition, calcium ion influx, hormone signaling, pathogenesis-related (PR) genes, transcription factors, and cell wall modification. In particular, the expression level of effector receptors (resistance proteins), PR genes involved in salicylic acid (SA) signaling pathway, were higher in clubroot-resistant NIL, while half of the PAMP receptors were suppressed in CR BJN3-2. This suggests that there was a more robust effector-triggered immunity (ETI) response in CR BJN3-2 and that SA signaling was important to clubroot resistance. The dataset generated by our transcriptome profiling may prove invaluable for further exploration of the different responses to *P. brassicae* between clubroot-resistant and clubroot-susceptible genotypes, and it will strongly contribute to a better understanding of the molecular mechanisms of resistance genes of *B. rapa* against *P. brassicae* infection.

## Introduction

Much research has focused on plant-pathogen interactions because of their importance to both agriculture and scientific research (Brown and Hovmoller, 2002). *Plasmodiophora brassicae* Wor. is a soil-borne, obligate, and biotrophic pathogen that attacks *Brassica* crops, leading to clubroot, and subsequent reductions in crop yield (Dixon, 2009). Many strategies have been proposed for controlling clubroot, among which the use of resistant cultivars is still the most cost-effective and environmentally friendly (Diederichsen et al., 2009; Donald and Porter, 2009).

Early reports suggested that both qualitative and quantitative traits were involved in clubroot resistance in *Brassica rapa*. At present, ten loci—*Crr1, Crr2, Crr3, Crr4, CRa, CRb, CRc, CRk, PbBa3.1*, and *PbBa3.3*—have been reported as being associated with clubroot resistance (CR) in *B. rapa* (Matsumoto et al., 1998; Suwabe et al., 2003, 2006; Hirai et al., 2004; Piao et al., 2004; Sakamoto et al., 2008; Chen et al., 2013). Two of these, *Crr1a* and *CRa*, have been cloned and found to be classical resistance genes that contain Toll-interleukin receptor (TIR)-nucleotide-binding (NB)-leucine-rich repeat (LRR; Ueno et al., 2012; Hatakeyama et al., 2013), but the response mechanisms to *P. brassicae* infection associated with these two CR genes have yet to be fully elucidated.

Plants have evolved two innate immune systems to combat with the attack of various pathogens (Jones and Dangl, 2006). The first mode of plant immune system is referred to as pathogen-associated molecular pattern (PAMP)-triggered immunity (PTI), which is triggered by the detection of PAMPs by pattern recognition receptor (PRR) proteins located on the external face of the host cell. However, pathogens can suppress PTI through secreting effectors into host cells. These pathogen effectors are recognized by specific resistance (*R*) genes in plants and triggers activation of the second mode of defense response, known as effector-triggered immunity (ETI). A number of defense responses are activated at the transcriptome level, including mitogen-activated protein kinase (MAPK) cascades, transcription factors, and hormone signaling (Moore et al., 2011), during PTI and ETI processes. Based on the transcriptome of *P. brassicae*, Schwelm et al. (2015) proposed that chitin might act as a PAMP of *P. brassicae* and the recognition of it can trigger defense responses in plants. However, these two processes have not been well characterized in *B. rapa* when challenged with *P. brassicae*.

*Brassica* crops are infected by *P. brassicae* in two distinct stages, consisting of primary infection of the root hairs followed by secondary infection of the root cortex (Kageyama and Asano, 2009). Feng et al. (2013) reported that primary and secondary infection of canola (*B. napus*) occurred 12 and 72 h, respectively, following inoculation with *P. brassicae*. The success of primary infection by *P. brassicae* was also observed in resistant genotypes of *Brassica* crops (Deora et al., 2012), indicating that in resistant *Brassica* strains *P*. *brassicae* is blocked at later stages of infection. Thus, studying the differentially expressed genes (DEGs) at two stages of infection helps in understanding host–*P. brassicae* interactions.

Analysis of global gene expression is one means of exploring the molecular basis of interactions between *Brassica* crops and *P. brassicae*, particularly with respect to mechanisms of resistance and the basal defense response. To date, several “-omics” approaches have been employed to examine the interactions between hosts and *P. brassicae*. Devos et al. (2006) and Cao et al. (2008), focusing on the changes of protein composition in *Arabidopsis* and *B. napus*, respectively, reported the details of the primary infection following inoculation. Their results demonstrated that most of the differentially regulated proteins were involved in plant defense, hormone metabolics, and detoxification. Siemens et al. (2006) identified more than 1000 DEGs in infected vs. control roots based on the ATH1 Affymetrix 22K microarray, which included genes associated with sugar phosphate metabolics, growth, cell cycle, and defense. A microarray analysis of *Arabidopsis thaliana* demonstrated that the number of DEGs involved in pathogen recognition and signal transduction was highest during the early stages of infection (Agarwal et al., 2011). A study using the complete *Arabidopsis* transcriptome microarray (CATMA) showed that, when compared with immune response in susceptible response, metabolic changes in the partial resistance response were reduced or delayed, and abnormal cell enlargement and proliferation were actively inhibited at 7 days post-inoculation on *A. thaliana* (Bur-0; Jubault et al., 2013). More recently, Schuller et al. (2014) confirmed the role of auxin and cytokinin metabolism and signaling in clubroot development based on microarray data and laser microdissection of *Arabidopsis* roots. They also found that brassinosteroid (BR) synthesis and signal perception were involved in clubroot development. Chu et al. (2014) reported that the signaling and metabolic activity of jasmonate acid (JA) and ethylene (ET) were up-regulated significantly in resistant populations compared to the susceptible lines at 15 days post-inoculation, while no increase in the expression of genes involved in salicylic acid (SA) metabolic and signaling pathways were detected. All of these functional studies focused on either the susceptible response or the late stage resistance response. Comparing responses to *P*. *brassicae* infection in susceptible and resistant plant lines is critical for understanding the defense mechanisms involved in resistance to clubroot.

In our previous studies, we identified and finely mapped a dominant *CRb* gene that confers resistance to *P*. *brassicae* pathotypes 2, 4, and 8 (Piao et al., 2004; Zhang et al., 2014). In addition, a pair of near-isogenic lines (NILs)—a clubroot-resistant (CR BJN3-2) line and a clubroot-susceptible (BJN3-2) line—were developed for *B. rapa* (Zhang et al., 2012a). The fact of similar genetic background and differences at the locus of a target gene between NILs would facilitate to analyze the response to pathogen in resistance and susceptible line as well as to compare the difference between the two lines on the transcription level.

RNA-Seq, a powerful approach for detecting DEGs and novel-expressed genes over a broad dynamic range (Blencowe et al., 2009; Wang et al., 2009b), was employed in this study: (i) to identify DEGs between CR BJN3-2 and BJN3-2; and (ii) to gain an insight into host–*P. brassicae* interactions for clubroot resistance during the early stages of infection by *P. brassicae*.

## Materials and methods

### Plant material

Two Chinese cabbage NILs carrying the clubroot-resistant allele of *CRbCRb* (CR BJN3-2) and the clubroot-susceptible allele of *crbcrb* (BJN3-2) were inoculated with suspension of *P. brassicae*. CR BJN3-2 was developed by integration of the *CRb* gene from the CR Shinki DH line of Chinese cabbage into the Chinese cabbage inbred line BJN3-2 based on the marker-assisted selection (Zhang et al., 2012a). SSR genotyping of CR BJN3-2 revealed that 100% of the recurrent parent genome was recovered.

### *P. brassicae* inoculation

The single-spore isolate (SSI) *P. brassicae*, which was used in the previous study (Zhang et al., 2014), was maintained and propagated in the susceptible Chinese cabbage lines. Resting spores were diluted to a density of 10^7^ spores per mL in sterile distilled water after isolation from homogenized clubbed roots. Thirty-day-old plants of CR BJN3-2 and BJN3-2 were inoculated with *P. brassicae* by injecting the soil around each plant with 1 mL of SSI suspension, in accordance with Piao et al. (2004). The inoculated plants were maintained in the culture room under a 16-h photoperiod at 25°C and the soil was kept moist during the treatment period.

### Microscopic investigation

To determine the timing of primary and secondary infection of the two NILs, infection processes of *P. brassicae* in the roots were observed using an inverted microscope (Olympus IX70, Japan) and imaged with digital single-lens reflex (DSLR) camera (Nikon D5300, Japan) every 2 h after inoculation until the secondary infection was established. The method of *P. brassicae* inoculation was the same as above. The roots were washed thoroughly with distilled water to remove the spores adsorbed on the surface, and then were transferred to a new 90 mm diameter glass petri dish with 30 ml distilled water for microscope observation. At each time point, five individual plants were examined, and three fields were observed for each sample.

### Tissue sampling

Based on the observed infection stages, the primary and secondary infection were found to be happened at 12 and 72 h after inoculation (hai). Therefore, the roots of the CR BJN3-2 and BJN3-2 plants were sampled at 0, 12, 72, and 96 hai for analysis of the DEGs at the early stages of infection. Three independent biological replicates of both two NILs were performed for *P. brassicae* inoculation. Roots of 30 (three biological replications, ten plants for each replicate) plants were sampled and pooled for RNA extraction at each time point. The roots were washed in distilled water to remove the pathogen inoculum and immediately frozen in liquid nitrogen and stored at −80°C until use. To verify the success of infection, a pair of NILs was maintained in the culture room for 30 days after inoculation.

To cover the shortage caused by the lack of biological replication for RNA-seq, three independent biological replications were performed for *P. brassicae* inoculation. Inoculation process and tissue sampling were exactly the same with the preparation of samples as above for RNA-seq. The pooled RNA was used to confirm the RNA-seq data results by quantitative RT-PCR (qRT-PCR).

### RNA isolation

RNA was extracted using TRIzol reagent (Invitrogen, Carlsbad, CA, USA) according to the manufacturer's instructions. RNA purity was determined using a Nanodrop spectrophotometer (Thermo Fisher Scientific Inc., Wilmington, DE, USA), 1% formaldehyde gel electrophoresis, and a 2100 Bioanalyzer (Agilent Technologies, Santa Clara, CA, USA). To eliminate the variability caused by human operation during the infection and sampling process, RNA from three biological replicates at each time point (0, 12, 72, and 96 hai) were pooled.

### cDNA library construction and sequencing

cDNA library preparation and sequencing were conducted by the Biomarker Technology Company in Beijing, China. mRNA was isolated using beads with Oligo (dT), and then broken into short fragments after the addition of fragmentation buffer. These short fragments of mRNA were used as templates to synthesize first-strand cDNA with random primers. Second-strand cDNA was synthesized in a reaction containing DNA polymerase I, RNase H, dNTPs, and buffer. The resulting cDNA were then subjected to end-repair polymerase and mixed with Solexa adapters. Suitable fragments were recovered from an agarose gel. Next, PCR amplification was performed to enrich the purified cDNA template. Finally, all the eight libraries (samples from 0, 12, 72, and 96 hai in two NILs) were sequenced using an Illumina HiSeq™ 2000. The raw RNA-seq data has been deposited at GEO (http://www.ncbi.nlm.nih.gov/geo/) under accession number GSE74044 (http://www.ncbi.nlm.nih.gov/geo/query/acc.cgi?acc=GSE74044).

### Digital gene expression analysis

Gene expression levels of all eight libraries were estimated by calculating read density as Reads Per Kilo base per Million mapped reads (RPKM; Mortazavi et al., 2008). EBseq software (Leng et al., 2013) was used to identify DEGs using pair-wise comparison. This program can estimate the variance of RNA-seq data without biological replicates by pooling similar genes together. *P*-values obtained via EBseq were further corrected using the Benjamini-Hochberg procedure (Benjamini and Hochberg, 1995), and the corrected *P*-values were used to determine the false discovery rate (FDR). Sequences were deemed to be significantly differentially expressed if the FDR was < 0.01 and there was at least a two-fold change (>1 or < −1 in log2 ratio value) in RPKM between two libraries. Differential patterns of gene expression at the various time points are represented by Venn diagrams.

### Sequence data analysis and annotation

We mapped sequencing reads to the reference database for the *B. rapa* genome (Version 1.2, http://brassicadb.org/brad/) using TopHat (Trapnell et al., 2009). Unigene sequences were compared with the nonredundant protein (nr) database (Deng et al., 2006), the Cluster of Orthologous Groups (COG) database (Tatusov et al., 2000), the Kyoto Encyclopedia of Genes and Genomes (KEGG) pathway database (Kanehisa et al., 2004), and Gene Ontology (GO; Ashburner et al., 2000). The Blast2GO program (Conesa et al., 2005) was used to obtain GO annotation of the unigenes. WEGO and Top GO software were then used to perform GO functional classification and enrichment analysis of all unigenes in order to view the distribution of gene functions.

### Quantitative RT-PCR analysis

To validate the RNA-seq results, we performed qRT-PCR on 40 selected DEGs. Twenty-one of the selected DEGs were involved in resistant to *P. brassicae* (including 9 receptor like proteins, 5 *R* genes, 2 calmodulin-like genes, 2 WRKY transcription factors, one beta-1,3 glucanase, and one cytochrome P450) and the rest of 19 DEGs were from the model of SA, JA, and ET signal pathway (Supplementary Table S1). Notably, 33 of the DEGs were from 0 hai.

The qRT-PCR reactions were performed using SYBR Green SuperReal PreMix Plus (TIANGEN) on a Bio-Rad CFX 96 real-time PCR system (Bio-Rad, Hercules, CA, USA). The primers were designed using Primer Premier 5.0 software and are listed in Supplementary Table S1. Analysis of gene expression was performed for all samples at 0, 12, 72, and 96 h after inoculation of CR BJN3-2 and BJN3-2 with *P*. *brassicae*. Each sample was divided into three independent biological and technical replicates. The comparative C_T_ method of quantitation was used with *B. rapa 18S rRNA* and *Actin* as internal controls. The conditions for amplification were as follows: 15 min denaturation at 95°C, followed by 40 cycles of 95°C for 10 s, 60°C for 20 s, and 72°C for 25 s. Following amplification, melting curves were performed by increasing temperatures from 55 to 95°C at intervals of 0.5°C every 10 s to confirm the specificity of the PCR amplification.

## Results

### Comparative analysis of *P. brassicae* infection of CR BJN3-2 and BJN3-2 genotypes

The differences in the infection process of *P. brassicae* between CR BJN3-2 and BJN3-2 were monitored after inoculation. There were no spores in the roots at 0 hai in both two lines, but spores were observed around the surface of the roots. Primary zoospores inside the root hairs were observed at 12 hai in both two lines. The presence of plasmodium in the cortical tissue was observed in BJN3-2 at 72 hai, indicating the beginning of secondary infection. Subsequently, numberous plasmodia were observed in the cortical tissue. In contrast, the secondary infection was not happened in CR BJN3-2 even after 72 and 96 hai. The same results were also obtained by Feng et al. (2013). Therefore, four time points (0, 12, 72, and 96 hai) were selected to investigate the differential transcript changes between two NILs at an early infection stage.

Severity of disease in both two NILs was assessed 30 days after inoculation with *P. brassicae* (Supplementary Figure S1). We found severe clubs on the roots of the susceptible line BJN3-2, but no clubroot symptoms were detected in the clubroot-resistant CR BJN3-2.

### RNA-seq analysis

Changes in transcript levels between CR BJN3-2 and BJN3-2 at 0, 12, 72, and 96 hai with *P. brassicae* were analyzed by RNA-seq. A total of 180,185,005 reads were generated by 100-bp paired-end sequencing from the eight cDNA libraries, constituting 35.4 Gb of cDNA sequence. Of these, 88,634,677 reads were obtained from CR BJN3-2 and 91,550,328 from BJN3-2. GC% of sequence data from the eight libraries were all ~48%, and Q30% (i.e., those reads with an average quality score >30) were all >80%, an indication that the accuracy and quality of the sequencing data were sufficient for further analysis. Approximately 78% of the sequenced reads (140 million mapped reads) were in alignment with the *B. rapa* genome reference sequence (Version 1.2, http://brassicadb.org). An overview of the sequencing process is shown in Supplementary Table S2. The distribution of the expression level of the unigenes had similar patterns among the eight samples, suggesting there was no bias in the construction of the cDNA libraries (Supplementary Figure S2).

The total mapped reads were aligned to each region in the reference genome, including the exon, intergenic, and intron regions, with the percentage of reads mapped to the exon being the highest in all eight libraries (Supplementary Figure S3).

### Transcriptome analysis in response to *P. brassicae*

**Figure 1:**
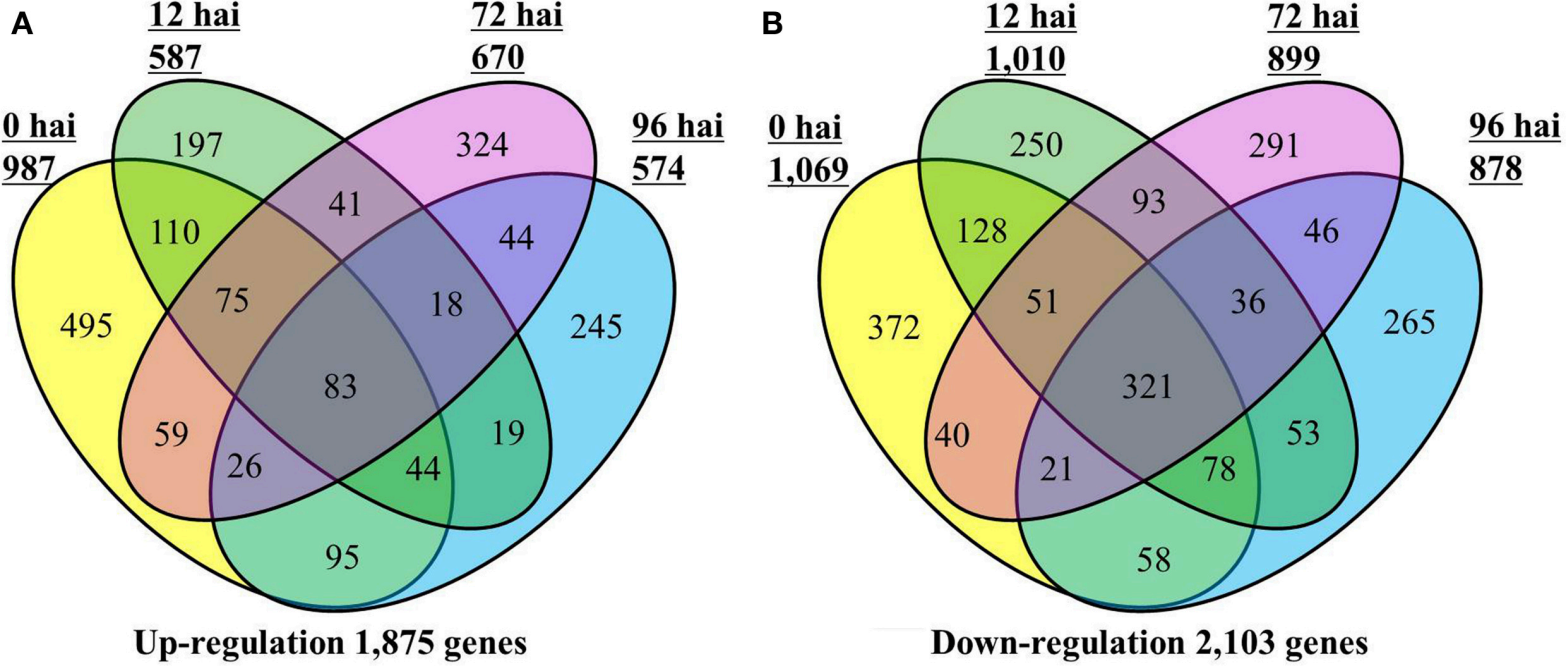
**Number of differentially expressed genes in the CR BJN3-2 that were (A) up-regulated and (B) down-regulated (FDR < 0.01 and fold change > 2.0 or < −2.0) compared with BJN3-2**. Numerals inside the parentheses indicate the number of genes expressed at each hai. The total number of DEGs is noted at the bottom of each Venn diagram. hai, hours after inoculation.

A total of 3812 DEGs were identified between CR BJN3-2 and BJN3-2 (Supplementary Table S3). As shown in the Venn diagram in Figure 1, 1875 genes were up-regulated and 2103 were down-regulated in CR BJN 3-2, while only 83 genes were up-regulated and 321 were down-regulated at all four time points. Compared with BJN 3-2, 987 genes were up-regulated and 1069 genes were down regulated at 0 hai. Following, *P. brassicae* inoculation, 587 genes were up-regulated, and 1010 genes were down-regulated at 12 hai; 670 gens were up-regulated and 899 were down-regulated at 72 hai; 574 genes were up-regulated and 878 genes were down-regulated at 96 hai. Number of the genes that were up/down-regulated was more than any other time point in CR BJN3-2 at 0 hai (Figure 1, Supplementary Tables S4–S5).

### Functional annotation of degs

All of the 3812 DEGs were annotated by comparing their sequences against five public databases, which permitted the assignment of several functional annotations (Supplementary Table S6): 3573 (93.73%) DEGs were found to have significant matches in the nr database, 2815 (73.85%) in the Swiss-Prot database, 1034 (27.12%) in the COG, 788 (20.67%) in the KEGG, and 3355 (88.01%) in the GO.

**Figure 2:**
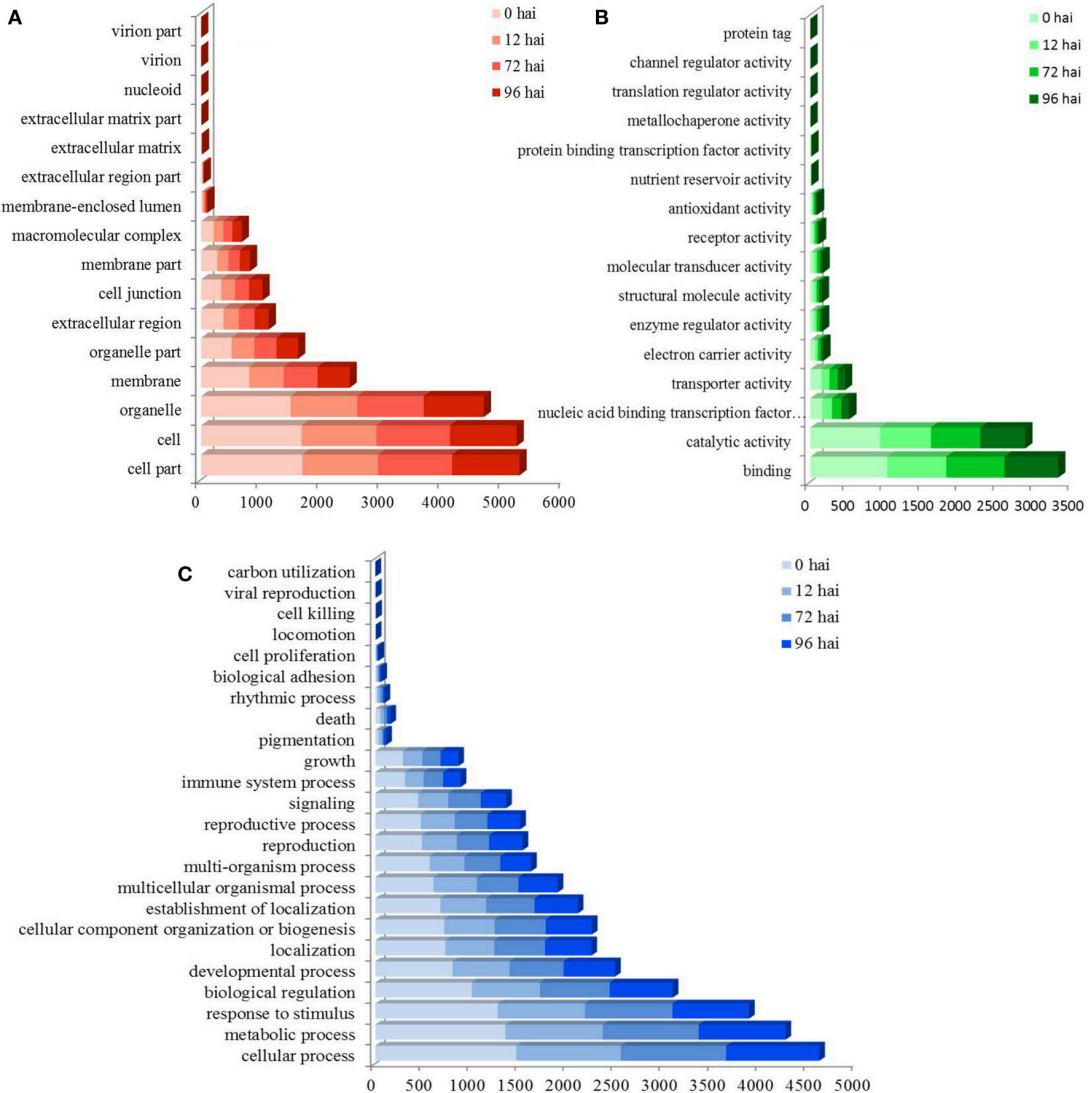
**GO assignment of differentially expressed genes (DEGs) in CR BJN3-2 and BJN3-2**. The unigenes were mapped to three main categories: cellular component **(A)**, molecular function **(B)**, and biological process **(C)**. The y-axis indicates the number of annotated DEGs. hai, hours after inoculation.

**Figure 3:**
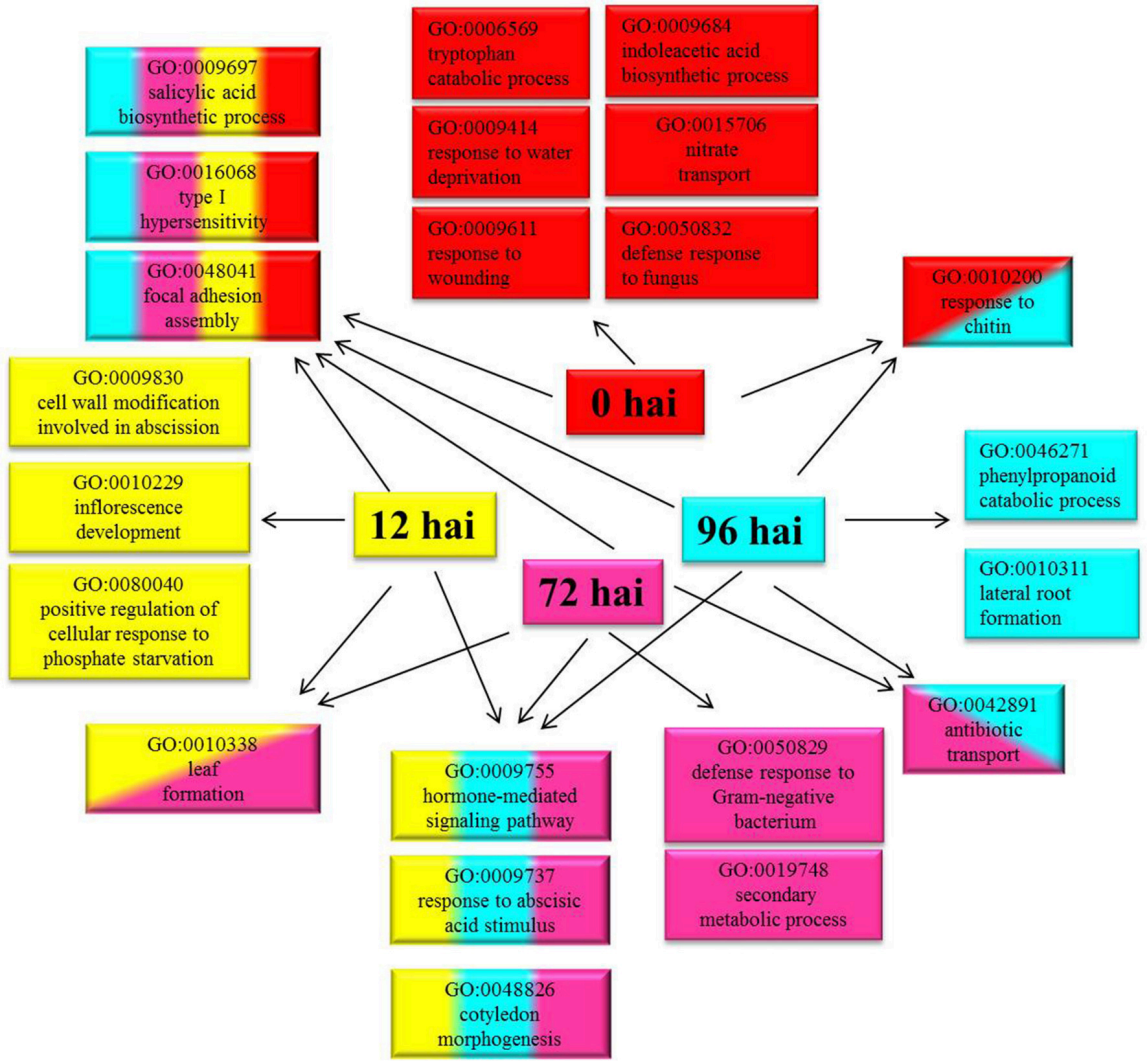
**Top ten Gene Ontology (GO) terms (biological process) that were significantly enriched at four time points**. GO terms enriched only at 0 hai are shown in red, GO terms enriched only at 12 hai are shown in yellow, GO terms enriched only at 72 hai are shown in purple, GO terms enriched only at 96 hai are shown in blue, and GO terms enriched at more than one time point are indicated in combined colors. hai, hour after inoculation.

A total of 3355 DEGs were assigned to three GO classes: biological process, cellular component, and molecular function. For the four time points, the cellular components most commonly represented were “cell” and “cell part,” whereas “catalytic activity” and “binding” were among the most commonly represented molecular function categories. The top five subcategories in the biological process class were “metabolic process,” “cellular process,” “response to stimulus,” “biological regulation,” and “developmental process” (Figure 2). GO enrichment analysis of the DEGs in the biological process class show the top GO terms at each time point represented as a directed acyclic graph (Supplementary Figures S4–S7). We extracted the top ten GO terms at each time point (Figure 3). For instance, the GO terms “salicylic acid biosynthetic process,” “type I hypersensitivity,” and “focal adhesion assembly” were present at all four time points. In total, there were 6, 3, 2, and 2 specific GO terms at 0, 12, 72, and 96 hai, respectively, (Figure 3).

**Figure 4:**
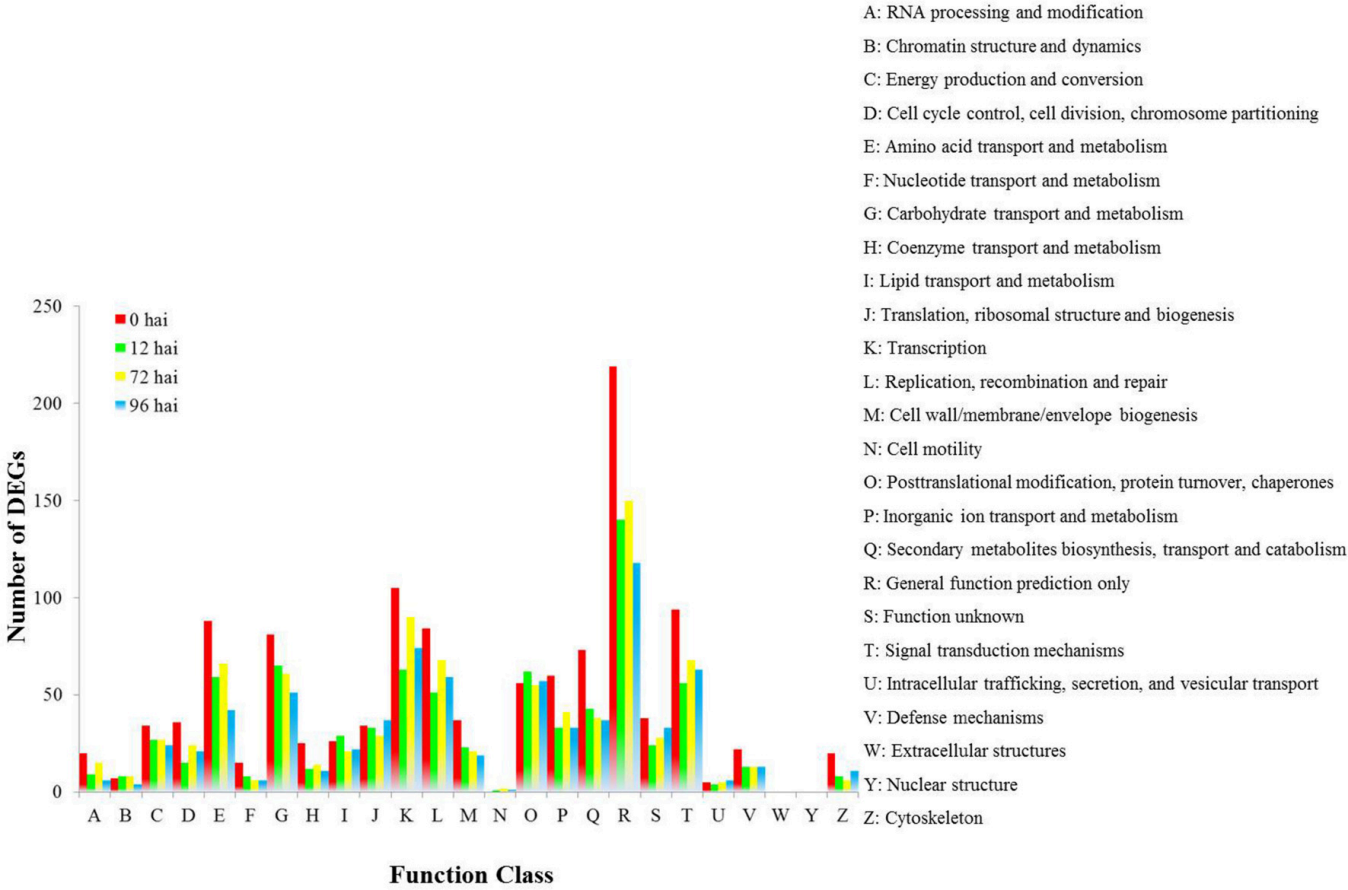
**COG functional classification of differentially expressed genes in CR BJN3-2 and BJN3-2**.

We found 1034 DEGs that had a COG classification. Among the 25 COG categories, the largest group was “General function prediction only” followed by “Tanscription,” “Signal transduction mechanisms,” “Amino acid transport and metabolism,” and “Replication, recombination and repair.” However, we did not find DEGs in the categories of W (Extracellular structure) and Y (Nuclear structure; Figure 4).

To identify the biological pathways that are active in *B. rapa*, we mapped the differentially expressed unigene sequences to the reference canonical pathways in KEGG. A total of 788 DEGs were annotated in the KEGG database and assigned to 111 KEGG pathways. The “Plant hormone signal transduction” was the most common term and contained 473 DEGs (12.41%), followed by “Ribosome” (448, 11.75%), “Plant–pathogen interaction” (283, 7.42%), “Oxidative phosphorylation” (265, 6.95%), and “Protein processing in endoplasmic reticulum” (258, 6.77%; Table 1).

**Table 1:** **Top 20 enriched KEGG pathways**.

**Pathway**	**The number of DEGs**	**Pathway ID**
Plant hormone signal transduction	473 (12.41%)	ko04075
Ribosome	448 (11.75%)	ko03010
Plant-pathogen interaction	283 (7.42%)	ko04626
Oxidative phosphorylation	265 (6.95%)	ko00190
Protein processing in endoplasmic reticulum	258 (6.77%)	ko04141
Spliceosome	255 (6.69%)	ko03040
Purine metabolism	235 (6.16%)	ko00230
RNA transport	231 (6.06%)	ko03013
Starch and sucrose metabolism	202 (5.30%)	ko00500
Glycolysis/Gluconeogenesis	193 (5.06%)	ko00010
Amino sugar and nucleotide sugar metabolism	189 (4.96%)	ko00520
Phenylpropanoid biosynthesis	185 (4.85%)	ko00940
Ubiquitin mediated proteolysis	185 (4.85%)	ko04120
Pyrimidine metabolism	184 (4.83%)	ko00240
Phenylalanine metabolism	160 (4.20%)	ko00360
mRNA surveillance pathway	157 (4.12%)	ko03015
Ribosome biogenesis in eukaryotes	150 (3.93%)	ko03008
Carbon fixation in photosynthetic organisms	146 (3.83%)	ko00710
Pyruvate metabolism	138 (3.62%)	ko00620
Arginine and proline metabolism	137 (3.59%)	ko00330

### DEGs involved in resistance to *P. brassicae*

**Figure 5:**
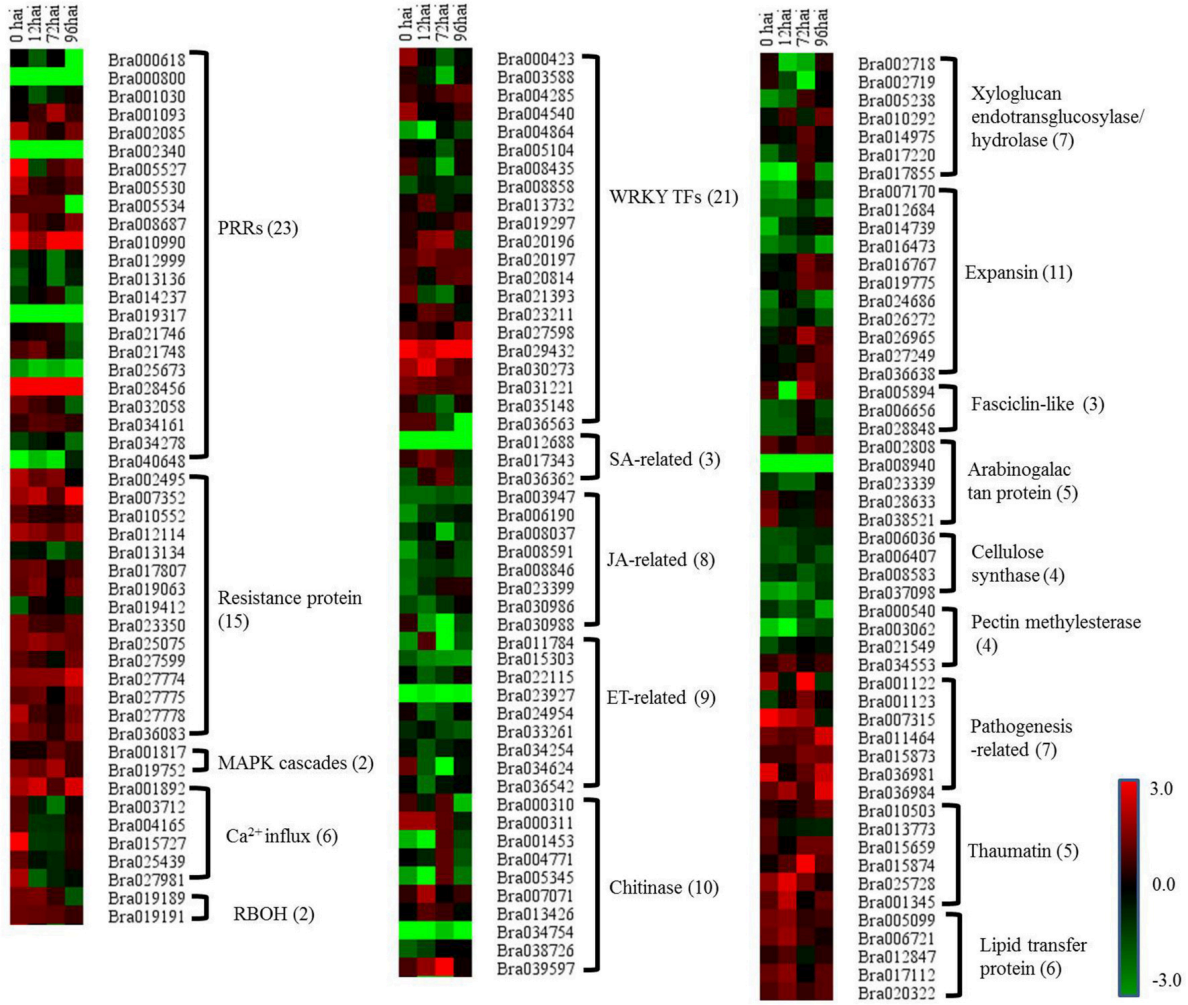
**Heat maps of differentially expressed genes for putative candidate genes assigned to clubroot-resistant genes in *B. rapa*.** R and S, resistant and susceptible genotype, respectively; hai, hours after inoculation; green indicates down-regulated DEGs, and red indicates up-regulated DEGs in CR BJN3-2 when compared to BJN3-2.

To gain a deeper insight into the defense mechanisms *B. rapa* employs to prevent infection by *P. brassicae*, a list of 151 differentially regulated disease resistance genes was obtained by combining data from the literature and a keyword search in the *B. rapa* genome annotation (Supplementary Table S7). These included 23 PRRs, 15 NBS-LRR containing *R* genes, 2 respiratory burst oxidase homologs (RBOH), 6 Ca^2+^ influx coding genes, 2 MAPK, 18 pathogenesis-related (PR) proteins, 20 hormone metabolism (SA [three genes], JA [eight genes], and ET [nine genes]) related genes, 34 cell wall modification related genes, 10 chitinases, and 23 WRKY factors. To get a global view of DEGs involved in resistant to *P. brassicae*, the fold changes of these genes between the two NILs were represented via a heat map (Figure 5). It is clearly that the *R* genes, MAPK, and PR proteins were up regulated in CR BJN3-2, while PRRs, WRKY factors and cell wall modification related genes showed an irregular regulation.

### Expression patterns of the genes in the SA, JA, and ET signaling pathways

In the current study, there were three genes related to the SA pathway, including two up-regulated *NIM1-interaction 2* (*NIMIN2*) genes, that were components of this defense signaling pathway (Figure 5, Supplementary Table S7). In addition, a *B. rapa* gene homolog to *SUPPRESSOR OF NPR1-1* was significantly down-regulated at all four time points (Figure 5, Supplementary Table S7), while eight genes involved in JA biosynthesis and signaling (including 12-oxophytodienoatereductase 1[*OPR1*], lipoxygenases 3 *[LOX3], LOX4*, jasmonate ZIM-domain [*JAZ*], and jasmonate-associated 1[*JAS1*]) and nine genes associated with ET biosynthesis and signaling (including 1-amino-cyclopropane-1-carboxylate synthase [*ACS*], ethylene-insensitive 3 [*EIN3*], EIN3-binding box protein [*EBF*], and ethylene response factor 2 [*ERF2*]) were down-regulated in CR BJN 3-2 at different time points (Figure 5, Supplementary Table S7). These results suggested an induced SA pathway and repressed JA/ET pathway during the host–*P. brassicae* interaction.

### Quantitative RT-PCR validation

**Figure 6:**
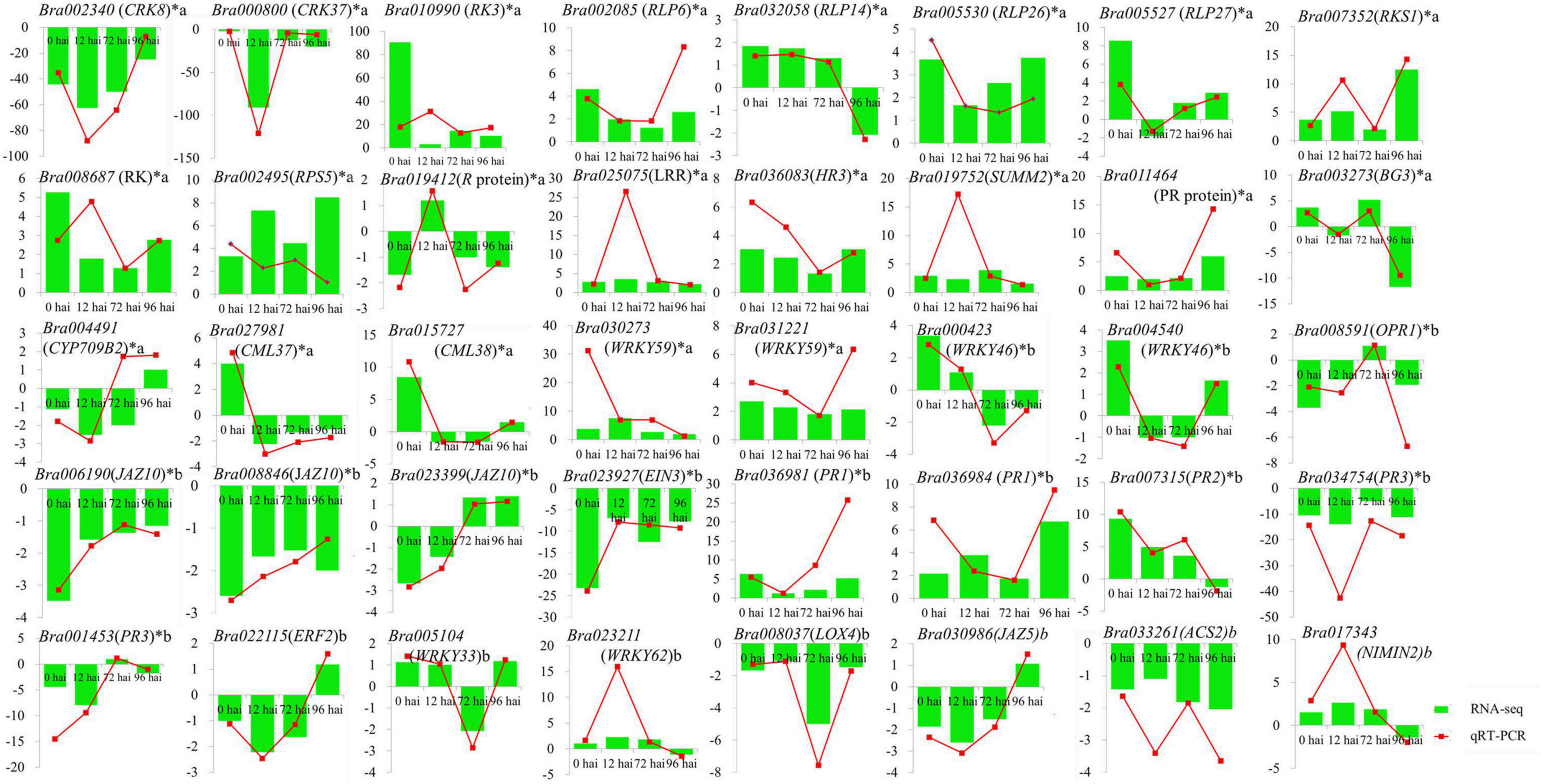
**Validation of RNA-seq data by qRT-PCR**. Forty DEGs involved in clubroot resistant and SA, JA, ET signal pathway (including 33 genes from 0 hai) were selected for validation and showed the same tendency with RNA-seq. y-axis showed the fold change between the two NILs, positive value indicated up regulated in CR BJN3-2 and negative indicated down regulated. ^*^indicated DEGs from 0 hai; a indicated DEGs involved in resistant to *P. brassicae*; b indicated DEGs from the model of SA, JA/ET signal pathway.

To confirm the results of the RNA-seq, 40 DEGs were selected for qRT-PCR assays, including 21 genes involved in resistant to *P. brassicae* and 19 genes from the model of SA, JA, and ET signal pathway (Supplementary Table S1). For all 40 DEGs, including 33 identified at 0 hai, qRT-PCR detected the same expression tendency as the RNA-seq analyses (Figure 6). It is a further indication of the high degree of reliability of the RNA-seq used in this study.

## Discussion

Identifying the genes that are regulated in response to *P*. *brassicae* infection represents a major challenge to understanding the basis of qualitative resistance. The use of a NIL pair carrying either the resistant or susceptible allele for the *CRb* locus in this study provided the opportunity to identify genes that exhibited differential transcript accumulation in the resistant or susceptible genotype. Compared to traditional genetic populations, NILs offer several advantages for transcriptional analyses due to the minimization of genetic background interference and enhancement of the sensitivity and accuracy of transcriptional analyses (Keurentjes et al., 2007). Although the RNA-seq data was analyzed without biological replicates, we could examine the *B. rapa* transcriptome at the early stages of *P. brassicae* infection, compares the response of susceptible and resistance lines of *B. rapa* to *P. brassicae* infection, and developed an integrated model for *B. rapa* during the early stages of *P. brassicae* infection.

A total of 3812 DEGs were detected between CR BJN3-2 and BJN3-2. Among them, more genes were detected to be differentially expressed at 0 hai. Detection of more DEGs at 0 hai were also found in potato and soybean when they were challenged by phytophthora and bacterial leaf pustule, respectively, (Kim et al., 2011; Gyetvai et al., 2012). However, it was not clear why more DEGs were detected at 0 hai. In the current study, DEGs related to the two main defense mechanisms (PTI and ETI) were investigated. One hundred and fifty one DEGs involved in PTI and ETI were found by combining data from the literature and a keyword search in the *B. rapa* genome annotation (Supplementary Table S7) and discussed below.

### Up-regulation of PR proteins in CR BJN3-2

One of the features of the defense response of plants is the production of PR proteins that are induced specifically in pathological or related situations (Van Loon and Van Strien, 1999). After inoculation with *P. brassicae*, 18 PR proteins (including 7 PR proteins, 5 thaumatin family proteins, and 6 lipid transfer proteins) were differentially regulated between CR BJN3-2 and BJN3-2 (Figure 5, Supplementary Table S7). All 18 PR proteins were up-regulated in CR BJN3-2 following inoculation with *P. brassicae*. This is consistent with a previous microarray analysis of *A. thaliana*, in which it was observed that several PR proteins, including thaumatin and defensins, were induced in the partially resistant line of the species (Jubault et al., 2013). Our results suggest that these PR proteins are most likely also involved in *B. rapa* resistance to *P. brassicae*.

### Pathogen perception by PRRs

The genes related to PRRs were induced in both two NILs. Among the 23 differentially expressed PRRs, 14 were induced in BJN3-2, and 9 were induced in CR BJN3-2 (Figure 5 and Supplementary Table S7). The initial step in the defense response of plants to the presence of a pathogen is the conserved PAMPs detected by PRRs, resulting in PTI (Jones and Dangl, 2006). PRRs thus play a fundamental role in PTI (Shiu and Bleecker, 2001). Of note, key PRR genes triggered by PAMPs, such as brassinosteroid insensitive 1-associated kinase 1 (*BAK1*), flagellin sensing 2 (*FLS2*), chitin elicitor receptor kinase (CERK), and chitin elicitor-binding protein (CEBiP), which transduces signals that trigger PTI (Dodds and Rathjen, 2010), showed no difference between CR BJN3-2 and BJN3-2, a finding that was contrary to our expectation. Using NILs of BLP-susceptible and BLP-resistant soybeans, Kim et al. (2011) examined the response of PTI-related genes after inoculation with *Xanthomonas axonopodis* pv. *glycines* and found that PRRs such as *FLS2* and *EFR* were up-regulated at 0 hai in the resistant soybean line. Li et al. (2012) proposed that *BAK1, CEBiP*, and *CERK1*, critical genetic components of PTI, were up-regulated following inoculation with *Foc TR4* in a resistant line of banana plant. That the PTI response in *B. rapa* as a result of *P. brassicae* infection differs from the response to other pathogens leads us to speculate that perhaps PTI does not play a role in the interaction between CR BJN 3-2 and *P. brassicae*.

### Pathogen detection by NB-LRR disease resistance protein

The NB-LRR containing *R* gene is a crucial component of ETI, as it detects and binds to pathogen effectors and triggers the subsequent defense response. In our study, of the 15 differentially expressed R proteins between the two NILs, only two genes encoding NBS-LRR proteins were down-regulated in CR BJN3-2 compared with BJN3-2 (Figure 5, Supplementary Table S7). Two transcripts homologous to *Arabidopsis* “*RESISTANT TO P. SYRINGAE 4*” (*RPS4*) and “*RESISTANT TO P. SYRINGAE 5*” (*RPS5*), respectively, were up-regulated at different time points in CR BJN3-2 (Supplementary Table S5). Previous work has shown that *RPS4* confers specific resistance to *Pseudomonas syringae* pv. *tomato* carrying the avirulence gene *AvrRPS4* (Gassmann et al., 1999). It was also reported that *RPS5* confers resistance to *P. syringae* strains that contain the avirulence gene *avrPphB* (Ade et al., 2007). Hence the *RPS4* and *RPS5* may be involved in the resistance interaction between *P. brassicae* and *B. rapa*. A *RPW8*-like gene encoding powdery mildew resistance protein (Wang et al., 2009a) was up-regulated in the *B. rapa* resistant NIL, as well. Taken together, our analysis suggests that the functions of *R* genes may be conserved across species and that the ETI response was more robust in CR BJN3-2 than in BJN3-2.

Previously, the *CRb* locus was narrowed to an interval of approximately 83.5 kb based on the “Chiifu-401-42” sequences located on chromosome A03 (Zhang et al., 2014). The target interval contained 15 putative genes and several classes of genes that could potentially be involved in resistance activities, including one TIR-NBS-LRR gene, one NBS–LRR gene, and several putative regulatory genes. There was, however, no significant difference in the expression of these genes between the two NILs (Supplementary Table S8). This may be due to the instability of the translation product and the differences in sequence between the CR regions of two NILs. Further confirmation via cloning of the *CRb* gene is needed.

### Activation of MAPK cascades and WRKY transcription factors

MAPK activation is one of the earliest signaling events following pathogen detection (Ichimura et al., 2002). Seven genes (*MPK3, MPK4, MPK6, MKK1, MKK2, MKK4*, and *MKK5*) were highly expressed at all time points in both NILs and no differences in the expression of these genes were found between CR BJN3-2 and BJN3-2 with the exception of *MKK5*, which was up-regulated at 72 hai in CR BJN3-2 (Figure 5, Supplementary Table S7). A *B. rapa* homolog of *SUPPRESSOR OF MKK1 MKK2 2* (*SUMM2*), a *R* protein encoding the coiled-coil (CC)-NB-LRR domain, was up-regulated at 0, 12, and 72 hai in CR BJN3-2. Zhang et al. (2012b) reported that *SUMM2* becomes active when the *MEKK1-MKK1*/*MKK2-MPK4* cascade is disrupted by pathogens, supporting the hypothesis that *R* proteins evolved to protect plants when microbial effectors suppress PTI. These results suggest that although PTI were suppressed in both NILs, ETI response was more robust in the resistant NIL. *WRKY*-type transcription factors, which act in a complex defense response network as both positive and negative regulators in plant immunity (Pandey and Somssich, 2009), were then activated by the MAPK cascade. In this study, some DEGs encoding *WRKY* exhibited different expression patterns between two NILs, with *WRKY 9*, −*16*, −*19*, −*29*, −*38*, −*46*, −*53*, −*59*, and −*62* being up-regulated in CR BJN3-2 at different time points whereas *WRKY 12*, −23, −33, −*40*, −*50*, and −*75* were down-regulated (Figure 5, Supplementary Table S7). It's unclear whether the *WRKY*s acted positively or negatively in response to the presence of *P. brassicae*, and thus the role of *WRKY* in clubroot resistance need further study.

### Ca^2+^ influx and respiratory burst oxidase homolog

The *B. rapa* calmodulin-like 37 (*CML 37*)-, *CML11-, CML24-*, and *CML38*-like genes were all up- regulated at 0 hai in CR BJN3-2 compared to BJN3-2 (Figure 5, Supplementary Table S7), indicating that Ca^2+^ participates in signal transduction during early-stage plant defense response. Similarly, an up-regulation of Ca^2+^-ATPase-like genes was also observed at 0 hai in soybean bacterial leaf pustule-resistance-NIL after inoculation with *X. axonopodis* pv. *glycines* (Kim et al., 2011). Calcium is an essential second messenger in the signal transduction pathways that regulate plant response to abiotic and biotic stress (Zipfel, 2009). Ca^2+^ can activate RBOH protein which was involved in the production of reactive oxygen species *in vitro* (Sagi and Fluhr, 2001). Two Chinese cabbage genes (*Bra019189* and *Bra019191*), the putative functions of which were RBOH, exhibited up-regulation in CR BJN3-2 (Figure 5, Supplementary Table S7). This result was in agreement with previous observations in wheat, cotton, and cucumber after infection by the fusarium wilt fungal pathogen (Dowd et al., 2004), and indicated that there was a higher ROS level in the resistant NILs, which inhibited the colonization of the pathogen on the roots.

### An induced SA signal pathway and repressed JA/ET signal pathway in resistant NIL

*P. brassicae* belongs to an obligate biotrophic protist. It is believed that plant resistance to biotrophic pathogens is controlled largely by SA-mediated signaling pathways, while resistance to necrotrophic pathogens is mediated by the JA and ET signaling pathways (Glazebrook, 2005). To study whether SA-mediated pathway was also involved in the resistant mechanism to *P. brassicae* in *B. rapa*, we examined the expression pattern of SA-related DEGs. It is found that NIMIN2, *PR1*, and *PR2* were up-regulated in the current study. NIMIN2 along with nonexpresser of PR genes 1 (*NPR1*) is a master regulator of the SA-mediated induction of defense genes (Spoel et al., 2003; Koornneef and Pieterse, 2008). Our results indicated an induced SA signal pathway in CR BJN3-2 after inoculation with *P. brassicae*. Consistently, Lovelock et al. (2013) demonstrated that SA is capable of suppressing clubroot in broccoli (*Brassicae oleracea* var. *italica*). And several studies have reported that *P. brassicae* secretes a methyl transferase (*PbBSMT*) that reduces the accumulation of SA in infected roots and that the expression level of *PbBSMT* in spores and plasmodia was low, whereas transferase expression was high in clubroots of host plants (Ludwig-Müller et al., 2015; Schwelm et al., 2015). All these studies suggested that a low SA level in the host can facilitate colonization by *P. brassicae*.

**Figure 7:**
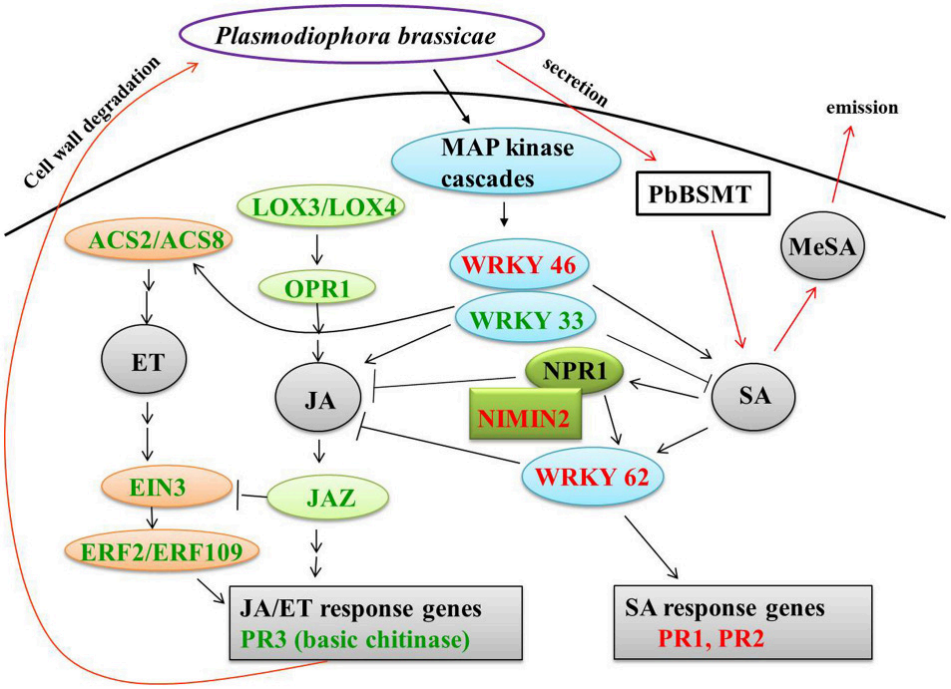
**Crosstalk between salicylic acid and jasmonate/ethylene**. Green and red coloring indicate down- and up-regulated genes, respectively, in CR BJN3-2. Black arrows, indicate results derived from the current study; red arrows, indicate results derived from other studies. ACS, 1-amino-cyclopropane-1-carboxylate synthase; EIN, ethylene-insensitive; ERF, ethylene response factor; JAZ, jasmonate-domain protein; LOX, lipoxygenase; MeSA, methyl salicylate; NIMIN, NIM1-interacting; NPR, nonexpresser of PR genes; OPR, 12-oxophytodienoate reductase; PbSMT, *P. brassicae* methyltransferase; PR, pathogenesis-related.

Eight genes related to JA biosynthesis and signaling and nine genes associated with ET biosynthesis and signaling were down-regulated in CR BJN3-2 (Figure 5, Supplementary Table S7). For example, *PR3* (basic chitinase) controlled by JA/ET-dependent pathways (Thomma et al., 1998) were down-regulated. The *WRKY62* was up-regulated in CR BJN3-2, which was demonstrated to function downstream of *NPR1* and regulate SA-mediated inhibition of JA signaling (Mao et al., 2007). The *JAZ* proteins can repress the function of *EIN3/EIL1*, probably by suppressing the DNA binding of *EIN3* (Zhu et al., 2011). Based on our results, a schematic illustration of the interactions between SA and JA (Figure 7) suggested that SA signal pathway was induced in the resistant NIL. Jubault et al. (2013) also reported the occurrence of an induced SA signaling pathway and a suppressed JA/ET signaling pathway in *A. thaliana* 7 dai with *P. brassicae*. Combined with previous results, it is clear that the SA signaling pathway and not the JA/ET signaling pathway plays a critical role in host resistance to *P*. *brassicae* infection.

### Cell wall modification

We also identified DEGs related to clubroot-induced abnormal cell enlargement and uncontrolled cell division of host roots. Siemens et al. (2006) reported that genes involved in cell division and expansion, such as cell cycle genes and expansins, were up-regulated in *A. thaliana* after inoculation with *P. brassicae*. Jubault et al. (2013) reported that cell enlargement and proliferation were actively inhibited in the partial quantitative resistance response compared to the susceptible one. Consistent with these studies, our analysis showed that most of cell division and expansion related genes were down regulated in resistant NIL. Of the 34 genes involved in cell division and expansion identified in the current study, only 11 genes were up-regulated in the clubroot-resistant NIL at 0, 12, 72, and 96 hai, including xyloglucan endotransglucosylase/hydrolase 16 and 24, expansin 7, 12 and 18, arabinogalactan protein (AGP) 1, 2 and 14, pectin methylesterase (PME) 44, with the remaining 23 genes being down-regulated in the resistant NIL (Figure 5, Supplementary Table S7). Cell division and expansion were suppressed in the clubroot-resistant NIL, indicating that the abnormal cell division that results from *P. brassicae* infection was suppressed in CR BJN 3-2.

### Chitinase in *B. rapa* response to *P. brassicae* infection

We identified 10 genes related to chitinase that were differentially expressed between the two NILs. Among them, 4 chitinase-related genes (*Bra007071, Bra013426, Bra000311*, and *Bra039597*) were up-regulated. Chitin is main component of the cell wall in *P. brassicae* (Moxham and Buczacki, 1983) and is known to be a kind of PAMP secreted by most pathogens (Latgé and Beauvais, 2014). It is not clear whether chitin released by *P. brassicae* is PAMP, but seven chitin synthase of *P. brassicae* were found to be highly expressed during infection process (Schwelm et al., 2015). The induced chitinase might provide the evidence for the degradation of chitin secreted by *P. brassicae* although it should be confirmed further.

Five genes were down-regulated in the CR BJN3-2 genotype compared to the BJN3-2 genotype, including three PR3-type basic chitinase (Figure 5, Supplementary Table S7). PR3 was controlled by JA/ET dependent pathway, and the induced PR3 exhibited the resistance to necrotrophic pathogens together with PR4 (chitinase type I and II) (Glazebrook, 2005). Down regulation of PR3 further indicated that the JA and ET pathways were not involved in the resistance to *P. brassicae* in *B. rapa*.

The chitinase gene *Bra004771* was up regulated at 72 hai, but down regulated at 96 hai in CR BJN3-2. There are also several reports showing that different types of chitinase operate against different pathogens in different plant species (Tabei et al., 1998; Takatsu et al., 1999; Datta et al., 2001; Rohini and Rao, 2001; Kishimoto et al., 2002; Takahashi et al., 2005). As such, the role that different chitinase subfamilies play in the interaction between *P. brassica* and *B. rapa* need to be investigated further.

## Conclusion

In this study, we first investigated the transcriptome response in the roots of clubroot-resistant and clubroot-susceptible NILs of *B*. *rapa* during the early stages of *P. brassicae* infection. A total of 3812 differentially expressed genes were identified. The up-regulation of resistance protein-coding genes, SA-related genes and PR genes demonstrated that these components play a role in the interaction between *B. rapa* and *P. brassicae*. At the same time, our results also showed that the genes involved in the uncontrolled cell division and expansion that are induced by clubroot were suppressed to a greater extent in resistant NIL than in susceptible NIL. The present work revealed that many genes are differentially expressed between clubroot-resistant and clubroot-susceptible NILs when responding to *P. brassicae* infection, greatly expanded the vision of the genetic basis and improved our understanding of the molecular mechanisms underlying *B. rapa* resistance to *P. brassicae*.

## Author contributions

JC analyzed the data, performed the experiments, and drafted the manuscript. WP participated in the data analysis and helped to draft the manuscript. BC helped analyzed the data. CZ contributed analysis tools and helped to draft the manuscript. ZP conceived the study, participated in its coordination, and helped to draft the manuscript. All authors have read and approved the final manuscript.

### Conflict of interest statement

The authors declare that the research was conducted in the absence of any commercial or financial relationships that could be construed as a potential conflict of interest.

## Supplementary material

Figure S1 **Disease symptoms in CR BJN3-2 and BJN3-2 30 days after *P. brassicae* inoculation**. There were no visible clubs on the CR BJN3-2 (left), but severe clubbing occurred on the main roots and lateral roots of BJN3-2 (right).

Figure S2 **Expression level distribution of all unigenes in eight libraries**. The figure shows that the expression level distribution pattern of unigenes among the eight samples were similar, suggesting there was no bias in the construction of the cDNA libraries.

Figure S3 **The percentage of Illumina sequencing reads mapped to reference genome regions in the eight libraries**. The total mapped reads were aligned to each region in the reference genome, including the exon, intergenic, and intron regions, in which the percentage of reads mapped to the exon was the highest in all eight libraries.

Figure S4 **The top GO directed acyclic graph of differentially expressed genes at 0 hai in biological process**.

Figure S5 **The top GO directed acyclic graph of differentially expressed genes at 12 hai in biological process**.

Figure S6 **The top GO directed acyclic graph of differentially expressed genes at 72 hai in biological process**.

Figure S7 **The top GO directed acyclic graph of differentially expressed genes at 96 hai in biological process**.
