# Supplementary material for: Transcriptome Analysis of Brassica rapa Near-Isogenic Lines Carrying Clubroot-Resistant and –Susceptible Alleles in Response to Plasmodiophora brassicae during Early Infection
Source: Front Plant Sci. 2016 Jan 5;6:1183. doi: 10.3389/fpls.2015.01183 (PMC4700149; doi:10.3389/fpls.2015.01183)
Supplement: Figure S1 — Disease symptoms in CR BJN3-2 and BJN3-2 30 days after P. brassicae inoculation. There were no visible clubs on the CR BJN3-2 (left), but severe clubbing occurred on the main roots and lateral roots of BJN3-2 (right). [file Presentation1.zip › Supplementary Material/Supplementary Table S1.docx]

***Supplementary Materials***

**Transcriptome analysis of *Brassica rapa* near-isogenic lines carrying clubroot-resistant and –susceptible alleles in response to *Plasmodiophora brassicae* during early infection**

**Jingjing Chen^1†^, Wenxing Pang^1†^, Bing Chen^2^, Chunyu Zhang^3*^ and Zhongyun Piao^1*^**

^†^Jingjing Chen and Wenxing Pang contributed equally to this work

*** Correspondence:**

Zhongyun Piao: zypiao@syau.edu.cn

Chunyu Zhang: zhchy@mail.hzau.edu.cn

**Supplementary Tables**

**Supplementary Table S1:** Gene information and primer sequence for qRT-PCR

| **Gene ID** | **Annotation** | **Forward primer 5’ 3’** | **Reverse primer 5’ 3’** |
| --- | --- | --- | --- |
| Bra002340 ^a^ | CRK8 | GATGCTACCGAGTTCAGA | TGTTCCTTTCACATACCG |
| Bra000800^a^ | CRK37 | GCTGCTTCTACGGCTGAC | CGACCAACTCCACCTCCT |
| Bra010990 ^a^ | RECEPTOR KINASE 2, RK2 | ATGAACCCGCTCAAGTCT | ACATTCCTCCAAACCCAC |
| Bra002085 ^a^ | RECEPTOR LIKE PROTEIN 6, RLP6 | GTTGCCATCTGATTACTT | GTGCCATTGATACTCCTTTA |
| Bra032058 ^a^ | RECEPTOR LIKE PROTEIN 14, RLP14 | GTTGGTTTGCTTGGCTTTA | AGGCTCCCGACAATAGGT |
| Bra005530 ^a^ | RECEPTOR LIKE PROTEIN 26, RLP26 | GCCCATACCCTATAACCA | CCACAAGGACCACAGTTT |
| Bra005527 ^a^ | RECEPTOR LIKE PROTEIN 27, RLP27 | CAGATTCTACGGTCCTAT | ATTTCGTTGTAGTCTCCC |
| Bra007352 ^a^ | RESISTANCE RELATED KINASE 1, RKS1 | TACCAGGTGAAGTATGTATCGG | GCAAAGTGGAGACGAGCA |
| Bra008687 ^a^ | receptor serine/threonine kinase, putative | AATGAAACTCGTGGACTC | CTTGACTTTGGCATAACT |
| Bra002495 ^a^ | RESISTANT TO P. SYRINGAE 5, RPS5 | AAGAGGAAAGCCAGAAGG | TTTGTTGGTGATGGGACT |
| Bra019412 ^a^ | disease resistance protein (TIR-NBS-LRR class) | ATCGGTCCCAAACTCATA | TCCTCACCTTTACCCTCA |
| Bra025075 ^a^ | leucine-rich repeat family protein | CAGCAGGAAGTGATGAAG | TTCGGACAGCAATCTATG |
| Bra036083 ^a^ | HOMOLOG OF RPW8 3, HR3 | ATGCCGATTGGTGAGGTT | TTCTGCGTCTGAGTTCTG |
| Bra019752 ^a^ | SUMM2, SUPPRESSOR OF MKK1 MKK2 2 | TGACAGGGTGGTGAAATG | AAATCCTTGAGACCGTTG |
| Bra011464 ^a^ | pathogenesis-related protein, putative | GAAATGTGGGTGAAGGAG | CGGGAGGATCATAGTTGC |
| Bra003273 ^a^ | BETA-1,3-GLUCANASE 3, BG3 | CGAGGCATTAGTGGGTTT | AGTGGACGGTGAAGTGAA |
| Bra004491 ^a^ | CYP709B2 | GGTATTGATTGGGTTCGT | CTGTTTGCTCCACTCCTC |
| Bra027981 ^a^ | CALMODULIN LIKE 37, CML37 | AACTGCGTCAGCCTCTTG | TACCGTCTTCTCCTTCCATC |
| Bra015727 ^a^ | CALMODULIN-LIKE 38, CML38 | CTTTCGTGAAGTTCTGTC | CAGCCTCTAAGTCTTTGTTC |
| Bra030273 ^a^ | WRKY59 | GAGAATCACTCACCCACCTT | TCGGTTTCTTGCCGTATT |
| Bra031221 ^a^ | WRKY59 | ACGGATACAAATGGAGGA | AGAAGGGCTTGGGTGGTT |
| Bra000423 ^ab^ | WRKY46 | TCCTCCGCATTTACCAGA | CTCCAGCAATAACCATCATCAA |
| Bra004540 ^ab^ | WRKY46 | AAGTGAAGTATGTCGGGAGC | CGTAGTCGCAGCAGGAGA |
| Bra008591 ^ab^ | OPR1 | ACTGACGAATACGGTGGA | AAGTGCTAACGCTTGTGG |
| Bra006190 ^ab^ | JAZ10 | CTTCGGTTCCGTCTACTC | GTCTTTCTTTGGTGCTGT |
| Bra008846 ^ab^ | JAZ10 | TAACCCTTCCGATACCTC | GAGACGGTCCCATTGTAG |
| Bra023399 ^ab^ | JAZ10 | ACGCTCCAAAGCCCAAGT | TAGCACCAGAAGCCAACA |
| Bra023927 ^ab^ | EIN3, ETHYLENE-INSENSITIVE3 | TGATTATGATTCCGCCAAGC | AAGCGAGCAACTCCCACC |
| Bra036981 ^ab^ | pathogenesis-related protein 1, putative | AACCCATCTCGAAACCTA | GTCTCGTCCCACGCTAAA |
| Bra036984 ^ab^ | pathogenesis-related protein 1, putative | CTCGCCGTGATGTTGGTG | ACGCCCGCCTGATTTAGT |
| Bra007315 ^ab^ | PATHOGENESIS-RELATED PROTEIN 2, PR2 | TGACGACCCATACTCTTACA | CACCACGATTTCCAACGAC |
| Bra034754 ^ab^ | BASIC CHITINASE,PR3 | AGTGTAACAGCCCGATTC | ATGGATGTGGGTATGACG |
| Bra001453 ^ab^ | BASIC CHITINASE, PR3 | AGCATCATTGGTCCTCGTC | CATGTTGAAGGTGGGTGG |
| Bra022115 ^b^ | ERF2, ETHYLENE RESPONSE FACTOR- 2 | GGTGGAGAAACCGAAGGC | CGTCCCTAACCAAACCCTC |
| Bra005104 ^b^ | WRKY33 | CAAACAGACTGGCACAAG | AGCGTAACCAATAGACCC |
| Bra023211 ^b^ | WRKY62 | ACAAACGACCAGAAATGC | TGTTGATGGGTAGAGGAAT |
| Bra008037 ^b^ | LIPOXYGENASE 4, LOX4 | ATAGACGCAACCTCCCACT | TAAACGGCTCCAAACACG |
| Bra030986 ^b^ | JAZ5 | AGCCAAGCCTGTGACTGA | CTGTGGGTTCGTTGAGAT |
| Bra033261 ^b^ | ACS2 | CTTGAGGTGGCGTACAGG | AAGACGGTGGCAGCATAG |
| Bra017343 ^b^ | NIM1-INTERACTING 2, NIMIN-2 | ACGGAGGAAGAGGTGGAT | GTAGCCCGACCCGATTTA |

**Note:** ^a^ indicated DEGs from 0 hai, ^b^ indicated DEGs from the model of SA, JA and ET signal pathway.
