# Supplementary material for: Transcriptome Analysis of Brassica rapa Near-Isogenic Lines Carrying Clubroot-Resistant and –Susceptible Alleles in Response to Plasmodiophora brassicae during Early Infection
Source: Front Plant Sci. 2016 Jan 5;6:1183. doi: 10.3389/fpls.2015.01183 (PMC4700149; doi:10.3389/fpls.2015.01183)
Supplement: Figure S1 — Disease symptoms in CR BJN3-2 and BJN3-2 30 days after P. brassicae inoculation. There were no visible clubs on the CR BJN3-2 (left), but severe clubbing occurred on the main roots and lateral roots of BJN3-2 (right). [file Presentation1.zip › Supplementary Material/Supplementary Table S2.docx]

***Supplementary Materials***

**Transcriptome analysis of *Brassica rapa* near-isogenic lines carrying clubroot-resistant and –susceptible alleles in response to *Plasmodiophora brassicae* during early infection**

**Jingjing Chen^1†^, Wenxing Pang^1†^, Bing Chen^2^, Chunyu Zhang^3*^ and Zhongyun Piao^1*^**

^†^Jingjing Chen and Wenxing Pang contributed equally to this work

*** Correspondence:**

Zhongyun Piao: zypiao@syau.edu.cn

Chunyu Zhang: zhchy@mail.hzau.edu.cn

**Supplementary Tables**

**Supplementary Table S2. Statistics of the sequenced reads and comparison to the *B. rapa* reference genome**

| Sample | Time Point  (hours after inoculation) | Total Reads | GC Percentage | Q30 Percentage | Unique Mapping Reads | Multi Mapping Reads | Mapped  Reads (%) |
| --- | --- | --- | --- | --- | --- | --- | --- |
| Resistant NIL | 0 | 45191092 | 48.17% | 82.80% | 34566592 | 800945 | 78.26% |
| (CR‘BJN3-2’) | 12 | 46364706 | 48.06% | 83.26% | 35638730 | 858701 | 78.72% |
|  | 72 | 38744502 | 48.14% | 82.46% | 29511904 | 706076 | 77.99% |
|  | 96 | 46969054 | 47.85% | 83.26% | 35993847 | 856350 | 78.46% |
| Susceptible NIL | 0 | 43490090 | 48.24% | 82.32% | 32990187 | 742356 | 77.56% |
| (‘BJN3-2’) | 12 | 45197498 | 48.07% | 82.84% | 34551936 | 800605 | 78.22% |
|  | 72 | 41472532 | 47.99% | 81.79% | 31111934 | 735970 | 76.79% |
|  | 96 | 52940536 | 47.72% | 82.98% | 40401873 | 953275 | 78.12% |
