# Supplementary material for: Transcriptome Analysis of Brassica rapa Near-Isogenic Lines Carrying Clubroot-Resistant and –Susceptible Alleles in Response to Plasmodiophora brassicae during Early Infection
Source: Front Plant Sci. 2016 Jan 5;6:1183. doi: 10.3389/fpls.2015.01183 (PMC4700149; doi:10.3389/fpls.2015.01183)
Supplement: Figure S1 — Disease symptoms in CR BJN3-2 and BJN3-2 30 days after P. brassicae inoculation. There were no visible clubs on the CR BJN3-2 (left), but severe clubbing occurred on the main roots and lateral roots of BJN3-2 (right). [file Presentation1.zip › Supplementary Material/Supplementary Table S6.docx]

***Supplementary Materials***

**Transcriptome analysis of *Brassica rapa* near-isogenic lines carrying clubroot-resistant and –susceptible alleles in response to *Plasmodiophora brassicae* during early infection**

**Jingjing Chen^1†^, Wenxing Pang^1†^, Bing Chen^2^, Chunyu Zhang^3*^ and Zhongyun Piao^1*^**

^†^Jingjing Chen and Wenxing Pang contributed equally to this work

*** Correspondence:**

Zhongyun Piao: zypiao@syau.edu.cn

Chunyu Zhang: zhchy@mail.hzau.edu.cn

**Supplementary Tables**

**Supplementary Table S6:** **Functional annotation of DEGs in five public databases**

| Time point | DEGs in nr | DEGs in SwissProt | DEGs in COG | DEGs in KEGG | DEGs in GO |
| --- | --- | --- | --- | --- | --- |
| 0 hai | 1931 | 1538 | 768 | 435 | 1805 |
| 12 hai | 1472 | 1175 | 544 | 321 | 1358 |
| 72 hai | 1449 | 1119 | 558 | 345 | 1336 |
| 96 hai | 1330 | 1035 | 504 | 306 | 1217 |
| total | 3573 | 2815 | 1034 | 788 | 3355 |
