# Supplementary material for: Transcriptome Analysis of Brassica rapa Near-Isogenic Lines Carrying Clubroot-Resistant and –Susceptible Alleles in Response to Plasmodiophora brassicae during Early Infection
Source: Front Plant Sci. 2016 Jan 5;6:1183. doi: 10.3389/fpls.2015.01183 (PMC4700149; doi:10.3389/fpls.2015.01183)
Supplement: Figure S1 — Disease symptoms in CR BJN3-2 and BJN3-2 30 days after P. brassicae inoculation. There were no visible clubs on the CR BJN3-2 (left), but severe clubbing occurred on the main roots and lateral roots of BJN3-2 (right). [file Presentation1.zip › Supplementary Material/Supplementary Table S7.docx]

***Supplementary Materials***

**Transcriptome analysis of *Brassica rapa* near-isogenic lines carrying clubroot-resistant and –susceptible alleles in response to *Plasmodiophora brassicae* during early infection**

**Jingjing Chen^1†^, Wenxing Pang^1†^, Bing Chen^2^, Chunyu Zhang^3*^ and Zhongyun Piao^1*^**

^†^Jingjing Chen and Wenxing Pang contributed equally to this work

*** Correspondence:**

Zhongyun Piao: zypiao@syau.edu.cn

Chunyu Zhang: zhchy@mail.hzau.edu.cn

**1. Supplementary Figures:**

**Supplementary Table S7:** List of putative resistance genes involved in clubroot resistant.

| **Functional Category** | | **Up/Down-**  **regulated** | | | **Gene** | | | | ***A. thaliana*** | | ***A. t* annotations** | | | **Log_2_ Fold Change** | | | | | | | | | |
| --- | --- | --- | --- | --- | --- | --- | --- | --- | --- | --- | --- | --- | --- | --- | --- | --- | --- | --- | --- | --- | --- | --- | --- |
|  |  |  |  |  |  |  |  |  |  |  |  |  |  | 0 hai 12 hai 72 hai 96 hai | | | | | | | | | |
| **PRRs** | | total of 23 genes | | | | | | | | | | | | | | | | | | | | | |
|  | | up | | Bra010990  Bra008687  Bra002085  Bra005530  Bra005527  Bra034161  Bra001093  Bra028456 | | | | AT1G65800.1 AT4G18250.1 AT1G45616.1 AT2G33050.1  AT2G33060.1 [AT3G05650.1](http://www.arabidopsis.org/servlets/TairObject?type=gene&id=37627)  AT4G04220.1  AT5G40380.1 | | | RECEPTOR KINASE2,RK2  Receptor serine/threonine kinase, putative  RECEPTOR LIKE PROTEIN 6, RLP6  RECEPTOR LIKE PROTEIN 26, RLP26  RECEPTOR LIKE PROTEIN 27, RLP27  RECEPTOR LIKE PROTEIN 32, RLP32  RECEPTOR LIKE PROTEIN 46, RLP46  CYSTEINE-RICH RLK 42,CRK42 | | 5.56  2.16  2.21  2.12  4.07  --  --  5.41 | | | | --  --  --  --  --  1.05  --  4.82 | | | 3.75  --  --  --  --  --  1.99  5.65 | --  1.52  1.39  --  --  --  --  5.91 | | |
|  | | down | | Bra025673 | | | | AT1G74190.1 | | | RECEPTOR LIKE PROTEIN 15, RLP15 | | -- | | | -- | | | | -2.1 | | -- | |
|  | |  | | Bra021748 | | | | AT2G32680.1 | | | RECEPTOR LIKE PROTEIN 23, RLP23 | | -- | | | -- | | | | -- | | -1 | |
|  | |  | | Bra005534 | | | | AT2G33020.1 | | | RECEPTOR LIKE PROTEIN 24, RLP24 | | -- | | | -- | | | | -- | | -3.2 | |
|  | |  | | Bra021746 | | | | AT2G33020.1 | | | RECEPTOR LIKE PROTEIN 24, RLP24 | | -- | | | -- | | | | -- | | -1.2 | |
|  | |  | | Bra014237 | | | | AT3G05360.1 | | | RECEPTOR LIKE PROTEIN 30, RLP30 | | -- | | | -- | | | | -- | | -1.5 | |
|  | |  | | Bra040648 | | | | AT3G05370.1 | | | RECEPTOR LIKE PROTEIN 31, RLP31 | | -- | | | -2.2 | | | | -2.7 | | -- | |
|  | |  | | Bra034278 | | | | AT3G05650.1 | | | RECEPTOR LIKE PROTEIN 32, RLP32 | | -- | | | -- | | | | -- | | -1.3 | |
|  | |  | | Bra000618 | | | | AT5G27060.1 | | | RECEPTOR LIKE PROTEIN 53, RLP53 | | -- | | | -- | | | | -- | | -3.8 | |
|  | |  | | Bra001030 | | | | AT3G02130.1 | | | RECEPTOR-LIKE PROTEIN KINASE 2 | | -- | | | -1.1 | | | | -- | | -- | |
|  | |  | | Bra012999 | | | | AT5G60900.1 | | | RECEPTOR-LIKE PROTEIN KINASE 1 | | -- | | | -- | | | | -1.5 | | -- | |
|  | |  | | Bra019317 | | | | AT4G23190.1 | | | RECEPTOR-LIKE PROTEIN KINASE 3 | | -9.1 | | | -7.5 | | | | -7 | | -7.9 | |
|  | |  | | Bra002340 | | | | AT4G23160.1 | | | CYSTEINE-RICH RLK 8,CRK8 | | -5.4 | | | -5.9 | | | | -5.4 | | -4.7 | |
|  | |  | | Bra000800 | | | | AT4G04500.1 | | | CYSTEINE-RICH RLK 37,CRK37 | | -5.7 | | | -6.4 | | | | -8.4 | | -4.1 | |
|  | |  | | Bra013136 | | | | AT2G13790.1 | | | SOMATIC EMBRYOGENESIS RECEPTOR-LIKE KINASE 4 | | -1.1 | | -- | | | | -1.4 | | | | -- |
|  | | Up/down | | Bra032058 | | | | AT1G74180.1 | | | RECEPTOR LIKE PROTEIN 14, RLP14 | | 1.33 | | -- | | | | -- | | | | -1.2 |
| **R proteins** | | total of 15 genes | | | | | | | | | | | | | | | | | | | | | |
|  | | up | Bra007352 | | | | [AT3G57710.1](http://www.arabidopsis.org/servlets/TairObject?type=gene&id=37346) | | | RESISTANCE RELATED KINASE 1, RKS1 | | | 1.85 | | 2.28 | | | -- | | | | | -- |
|  | |  | Bra027599 | | | | [AT5G45250.1](http://www.arabidopsis.org/servlets/TairObject?type=gene&id=432775) | | | RESISTANT TO P.SYRINGAE 4, RPS4 | | | 1.09 | | -- | | | -- | | | | | 1.36 |
|  | |  | Bra002495 | | | | [AT1G12220.1](http://www.arabidopsis.org/servlets/TairObject?type=gene&id=138638) | | | RESISTANT TO P. SYRINGAE 5, RPS5 | | | 2.12 | | 1.18 | | | 1.56 | | | | | -- |
|  | |  | Bra036083 | | | | [AT3G50470.1](http://www.arabidopsis.org/servlets/TairObject?type=gene&id=41209) | | | HOMOLOG OF RPW8 3, HR3 | | | 1.62 | | 1.26 | | | -- | | | | | -- |
|  | |  | Bra025075 | | | | [AT5G45510.1](http://www.arabidopsis.org/servlets/TairObject?type=gene&id=134600) | | | leucine-rich repeat family protein | | | 1.5 | | 1.82 | | | 1.43 | | | | | 1.15 |
|  | |  | Bra012114 | | | | [AT5G66900.1](http://www.arabidopsis.org/servlets/TairObject?type=gene&id=136131) | | | disease resistance protein (CC-NBS-LRR class) | | | 1.99 | | 1.66 | | | 1.14 | | | | | 1.72 |
|  | |  | Bra019063 | | | | [AT4G27190.1](https://www.arabidopsis.org/servlets/TairObject?type=gene&id=131010) | | | disease resistance protein (NBS-LRR class) | | | -- | | 1.54 | | | -- | | | | | 1.28 |
|  | |  | Bra027774 | | | | [AT1G63880.1](https://www.arabidopsis.org/servlets/TairObject?type=gene&id=137693) | | | disease resistance protein (TIR-NBS-LRR class) | | | -- | | 1.66 | | | 1.65 | | | | | -- |
|  | |  | Bra027778 | | | | [AT1G63880.1](https://www.arabidopsis.org/servlets/TairObject?type=gene&id=137693) | | | disease resistance protein (TIR-NBS-LRR class) | | | 2.13 | | -- | | | -- | | | | | 1.32 |
|  | |  | Bra023350 | | | | [AT5G36930.2](https://www.arabidopsis.org/servlets/TairObject?type=gene&id=1000652046) | | | disease resistance protein (TIR-NBS-LRR class) | | | 1.49 | | -- | | | -- | | | | | 1.24 |
|  | |  | Bra017807 | | | | [AT2G14080.1](https://www.arabidopsis.org/servlets/TairObject?type=gene&id=431855) | | | disease resistance protein (TIR-NBS-LRR class) | | | 1.22 | | 1.35 | | | -- | | | | | -- |
|  | |  | Bra027775 | | | | [AT1G63870.1](https://www.arabidopsis.org/servlets/TairObject?type=gene&id=137695) | | | disease resistance protein (TIR-NBS-LRR class) | | | 1.13 | | 1.27 | | | -- | | | | | 1.67 |
|  | |  | Bra010552 | | | | [AT4G36150.1](https://www.arabidopsis.org/servlets/TairObject?type=gene&id=128959) | | | disease resistance protein (TIR-NBS-LRR class) | | | 1.06 | | -- | | | -- | | | | | -- |
|  |  | down | Bra013134 | | | | AT1G58410.1 | | | disease resistance protein (CC-NBS-LRR class) | | | -- | | -- | | | -1.3 | | | | | -- |
|  |  |  | Bra019412 | | | | AT5G11250.1 | | | disease resistance protein (TIR-NBS-LRR class) | | | -1.2 | | -- | | | -- | | | | | -- |
| **MAPK cascade and WRKY TFs** | | | | | | total of 23 genes | | | | | | | | |  | | |  | | | | |  |
| MAPK cascades | up | | Bra001817 | | | | [AT3G21220.1](http://www.arabidopsis.org/servlets/TairObject?type=gene&id=40531) | | | MITOGEN-ACTIVATED PROTEIN KINASE KINASE 5, MKK5 | | -- | | | -- | | | 1.13 | | | | | -- |
|  |  | | Bra019752 | | | | [AT1G12280.1](http://www.arabidopsis.org/servlets/TairObject?type=gene&id=31966) | | | SUMM2, SUPPRESSOR OF MKK1 MKK2 2 | | 1.56 | | | 1.23 | | | 1.97 | | | | | -- |
| WRKY TFs | up | | Bra004285 | | | | [AT1G68150.1](https://www.arabidopsis.org/servlets/TairObject?type=gene&id=138252) | | | ATWRKY9, WRKY DNA-BINDING PROTEIN 9 | | -- | | | -- | | | -- | | | | | 1.24 |
|  |  | | Bra027598 | | | | [AT5G45050.1](https://www.arabidopsis.org/servlets/TairObject?type=gene&id=133446) | | | ATWRKY16, WRKY16 | | 1.08 | | | -- | | | -- | | | | | 1.67 |
|  |  | | Bra029432 | | | | [AT4G12020.2](https://www.arabidopsis.org/servlets/TairObject?type=gene&id=1000651624) | | | ATWRKY19, WRKY19 | | 5.38 | | | -- | | | 3.13 | | | | | 5.42 |
|  |  | | Bra019297 | | | | [AT4G23550.1](http://www.arabidopsis.org/servlets/TairObject?type=gene&id=129838) | | | ATWRKY29, WRKY29 | | -- | | | -- | | | -- | | | | | 1.02 |
|  |  | | Bra020814 | | | | [AT4G23550.1](http://www.arabidopsis.org/servlets/TairObject?type=gene&id=129838) | | | ATWRKY29, WRKY29 | | -- | | | -- | | | -- | | | | | 1.15 |
|  |  | | Bra020196 | | | | [AT5G22570.1](https://www.arabidopsis.org/servlets/TairObject?type=gene&id=135577) | | | ATWRKY38,WRKY38 | | -- | | | 1.6 | | | 1.85 | | | | | -- |
|  |  | | Bra020197 | | | | [AT5G22570.1](https://www.arabidopsis.org/servlets/TairObject?type=gene&id=135577) | | | ATWRKY38, WRKY38 | | -- | | | 1.43 | | | 1.04 | | | | | -- |
|  |  | | Bra004540 | | | | [AT2G46400.1](https://www.arabidopsis.org/servlets/TairObject?type=gene&id=32663) | | | ATWRKY46, WRKY46 | | 1.83 | | | -- | | | -- | | | | | -- |
|  |  | | Bra013732 | | | | [AT4G23810.1](https://www.arabidopsis.org/servlets/TairObject?type=gene&id=432541) | | | ATWRKY53, WRKY53 | | -- | | | 1.15 | | | -- | | | | | -- |
|  |  | | Bra031221 | | | | [AT2G21900.1](http://www.arabidopsis.org/servlets/TairObject?type=gene&id=34714) | | | ATWRKY59, WRKY59 | | 1.46 | | | 1.12 | | | -- | | | | | 1.01 |
|  |  | | Bra030273 | | | | [AT2G21900.1](https://www.arabidopsis.org/servlets/TairObject?type=gene&id=34714) | | | ATWRKY59, WRKY59 | | 1.91 | | | 2.85 | | | 1.42 | | | | | -- |
|  | | Bra023211 | | | | [AT5G01900.1](http://www.arabidopsis.org/servlets/TairObject?type=gene&id=136952) | | | ATWRKY62, WRKY62 | | -- | | | 1.17 | | | -- | | | | | -- |  |
| down | | Bra004864 | | | | [AT2G44745.1](https://www.arabidopsis.org/servlets/TairObject?type=gene&id=434686) | | | ATWRKY12, WRKY12 | | -- | | | -4.1 | | | -- | | | | | -- |  |
|  |  | | Bra005104 | | | | [AT2G38470.1](http://www.arabidopsis.org/servlets/TairObject?type=gene&id=35461) | | | ATWRKY33, WRKY33 | | -- | | | -- | | | -1.1 | | | | | -- |
|  |  | | Bra003588 | | | | [AT1G80840.1](https://www.arabidopsis.org/servlets/TairObject?type=gene&id=30510) | | | ATWRKY40, WRKY40 | | -- | | | -- | | | -2.2 | | | | | -- |
|  |  | | Bra008435 | | | | [AT1G80840.1](https://www.arabidopsis.org/servlets/TairObject?type=gene&id=30510) | | | ATWRKY40, WRKY40 | | -- | | | -- | | | -2.1 | | | | | -- |
|  |  | | Bra035148 | | | | [AT1G80840.1](https://www.arabidopsis.org/servlets/TairObject?type=gene&id=30510) | | | ATWRKY40, WRKY40 | | -- | | | -- | | | -1.3 | | | | | -- |
|  |  | | Bra036563 | | | | [AT5G26170.1](https://www.arabidopsis.org/servlets/TairObject?type=gene&id=136768) | | | ATWRKY50, WRKY50 | | -- | | | -- | | | -- | | | | | -3.7 |
|  |  | | Bra008858 | | | | [AT5G13080.1](https://www.arabidopsis.org/servlets/TairObject?type=gene&id=136781) | | | ATWRKY75, WRKY75 | | -1 | | | -- | | | -- | | | | | -- |
|  | up/down | | Bra021393 | | | | [AT2G47260.1](https://www.arabidopsis.org/servlets/TairObject?type=gene&id=36728) | | | ATWRKY23, WRKY23 | | 1.19 | | | -- | | | -1.4 | | | | | -- |
|  |  | | Bra000423 | | | | [AT2G46400.1](https://www.arabidopsis.org/servlets/TairObject?type=gene&id=32663) | | | ATWRKY46, WRKY46 | | 1.77 | | | -- | | | -1.1 | | | | | -- |
| **Ca^2+^ influx and RBOH** | | | total of 8 genes | | | | | | |  | |  | | |  | | |  | | | | |  |
| Ca^2+^ influx | up | | Bra001892 | | | | [AT3G22930.1](https://www.arabidopsis.org/servlets/TairObject?type=gene&id=38743) | | | CALMODULIN-LIKE 11, CML11 | | 2 | | | 2.53 | | | -- | | | | | 2.62 |
|  |  | | Bra025439 | | | | [AT5G37770.1](https://www.arabidopsis.org/servlets/TairObject?type=gene&id=433378) | | | CALMODULIN-LIKE 24, CML24 | | 1.19 | | | -- | | | -- | | | | | -- |
|  |  | | Bra004165 | | | | [AT5G37770.1](https://www.arabidopsis.org/servlets/TairObject?type=gene&id=433378) | | | CALMODULIN-LIKE 24, CML24 | | 1.09 | | | -- | | | -- | | | | | -- |
|  |  | | Bra015727 | | | | [AT1G76650.1](https://www.arabidopsis.org/servlets/TairObject?type=gene&id=31239) | | | CALMODULIN-LIKE 38, CML38 | | 3.09 | | | -- | | | -- | | | | | -- |
|  |  | | Bra003712 | | | | [AT1G76650.1](https://www.arabidopsis.org/servlets/TairObject?type=gene&id=31239) | | | CALMODULIN-LIKE 38, CML38 | | 1.05 | | | -- | | | -1.4 | | | | | -- |
|  | up/down | | Bra027981 | | | | [AT5G42380.1](http://www.arabidopsis.org/servlets/TairObject?type=gene&id=134436) | | | CALMODULIN LIKE 37, CML37 | | 2.02 | | | -1.2 | | | -- | | | | | -- |
| RBOH | up | | Bra019191 | | | | [AT4G25090.1](https://www.arabidopsis.org/servlets/TairObject?type=gene&id=432296) | | | respiratory burst oxidase, putative / NADPH oxidase, putative | | 1.27 | | | 1.19 | | | \| 1.07 \| \| --- \| | | | | | -- |
|  | up/down | | Bra019189 | | | | [AT4G25090.1](https://www.arabidopsis.org/servlets/TairObject?type=gene&id=432296) | | | respiratory burst oxidase, putative / NADPH oxidase, putative | | 1.36 | | | 1.42 | | | -- | | | | | -1 |
| **Hormone metabolism** | | | total of 20 genes | | | | | | |  | |  | | |  | | |  | | | | |  |
| Salicylic acid | | up | Bra017343 | | | | [AT3G25882.1](http://www.arabidopsis.org/servlets/TairObject?type=gene&id=500439935) | | | NIM1-INTERACTING 2, NIMIN-2 | | -- | | | -1.39 | | | -- | | | | | -- |
|  | |  | Bra036362 | | | | [AT3G25882.1](http://www.arabidopsis.org/servlets/TairObject?type=gene&id=500439935) | | | NIM1-INTERACTING 2, NIMIN-2 | | -- | | | -- | | | -1.28 | | | | | -- |
|  | | down | Bra012688 | | | | [AT4G16890.1](https://www.arabidopsis.org/servlets/TairObject?type=gene&id=129967) | | | SUPPRESSOR OF NPR1-1, CONSTITUTIVE 1 | | -9 | | | -9.9 | | | -9.1 | | | | | -7.1 |
| Jasmonic acid | | down | Bra008591 | | | | [AT1G76680.2](http://www.arabidopsis.org/servlets/TairObject?type=gene&id=1000644048) | | | 12-OXOPHYTODIENOATE REDUCTASE 1,OPR1 | | -1.9 | | | -- | | | -- | | | | | -- |
|  |  |  | Bra008037 | | | | [AT1G72520.1](http://www.arabidopsis.org/servlets/TairObject?type=gene&id=31251) | | | LIPOXYGENASE 4, LOX4 | | -- | | | -- | | | -2.3 | | | | | -- |
|  | |  | Bra030988 | | | | [AT1G17420.1](http://www.arabidopsis.org/servlets/TairObject?type=gene&id=434296) | | | LIPOXYGENASE 3, LOX3 | | -- | | | -- | | | -5.6 | | | | | -- |
|  | |  | Bra003947 | | | | [AT1G70700.1](http://www.arabidopsis.org/servlets/TairObject?type=gene&id=31789) | | | JASMONATE-ZIM-DOMAIN PROTEIN 9 | | -1.3 | | | -- | | | -- | | | | | -- |
|  | |  | Bra006190 | | | | [AT5G13220.1](http://www.arabidopsis.org/servlets/TairObject?type=gene&id=137329) | | | JASMONATE-ZIM-DOMAIN PROTEIN 10 | | -1.8 | | | -- | | | -- | | | | | -- |
|  | |  | Bra008846 | | | | [AT5G13220.1](http://www.arabidopsis.org/servlets/TairObject?type=gene&id=137329) | | | JASMONATE-ZIM-DOMAIN PROTEIN 10 | | -1.3 | | | -- | | | -- | | | | | -- |
|  | |  | Bra030986 | | | | [AT1G17380.1](http://www.arabidopsis.org/servlets/TairObject?type=gene&id=434301) | | | JASMONATE-ZIM-DOMAIN PROTEIN 5 | | -- | | | -1.4 | | | -- | | | | | -- |
|  | |  | Bra023399 | | | | [AT5G13220.1](http://www.arabidopsis.org/servlets/TairObject?type=gene&id=137329) | | | JASMONATE-ASSOCIATED 1, JASMONATE-ZIM-DOMAIN PROTEIN 10 | | -1.4 | | | -- | | | -- | | | | | -- |
| Ethylene | | down | Bra033261 | | | | [AT1G01480.1](http://www.arabidopsis.org/servlets/TairObject?type=gene&id=30449) | | | 1-AMINO-CYCLOPROPANE-1-CARBOXYLATE SYNTHASE 2, ACS2 | | -- | | | -- | | | -- | | | | | -1.1 |
|  | |  | Bra011784 | | | | [AT4G37770.1](http://www.arabidopsis.org/servlets/TairObject?type=gene&id=131154) | | | 1-AMINO-CYCLOPROPANE-1-CARBOXYLATE SYNTHASE 8, ACS8 | | -- | | | -- | | | -3.4 | | | | | -- |
|  | |  | Bra023927 | | | | [AT3G20770.1](http://www.arabidopsis.org/servlets/TairObject?type=gene&id=40048) | | | EIN3, ETHYLENE-INSENSITIVE3 | | -4.4 | | | -2.7 | | | -3.6 | | | | | -2.9 |
|  | |  | Bra034254 | | | | [AT2G25490.1](http://www.arabidopsis.org/servlets/TairObject?type=gene&id=32833) | | | EBF1, EIN3-BINDING F BOX PROTEIN 1 | | -- | | | -1 | | | -- | | | | | -- |
|  | |  | Bra036542 | | | | [AT5G25350.1](http://www.arabidopsis.org/servlets/TairObject?type=gene&id=132131) | | | EBF2, EIN3-BINDING F BOX PROTEIN 2 | | -- | | | -1.3 | | | -- | | | | | -- |
|  | |  | Bra022115 | | | | [AT5G47220.1](http://www.arabidopsis.org/servlets/TairObject?type=gene&id=135628) | | | ERF2, ETHYLENE RESPONSE FACTOR- 2 | | -- | | | -1.2 | | | -- | | | | | -- |
|  | |  | Bra034624 | | | | [AT4G34410.1](http://www.arabidopsis.org/servlets/TairObject?type=gene&id=128090) | | | ETHYLENE RESPONSE FACTOR 109 | | -- | | | -- | | | -3.4 | | | | | -- |
|  | |  | Bra015303 | | | | [AT1G04310.1](http://www.arabidopsis.org/servlets/TairObject?type=gene&id=29700) | | | ETHYLENE RESPONSE SENSOR 2 | | -- | | | -1.6 | | | -1.8 | | | | | -1.9 |
|  | |  | Bra024954 | | | | [AT5G47220.1](http://www.arabidopsis.org/servlets/TairObject?type=gene&id=135628) | | | ETHYLENE RESPONSIVE ELEMENT BINDING FACTOR 2 | | -- | | | -1.3 | | | -- | | | | | -- |
| **cell wall modification** | | | total of 34 genes | | | | | | |  | |  | | |  | | |  | | | | |  |
| xyloglucan endotransglucosylase/  hydrolase | | up | Bra014975 | | | | [AT3G23730.1](http://www.arabidopsis.org/servlets/TairObject?type=gene&id=40600) | | | XYLOGLUCAN ENDOTRANSGLUCOSYLASE/  HYDROLASE 16 | | -- | | | -- | | | 1.1 | | | | | -- |
|  |  |  | Bra010292 | | | | [AT4G30270.1](http://www.arabidopsis.org/servlets/TairObject?type=gene&id=129916) | | | XYLOGLUCAN ENDOTRANSGLUCOSYLASE/  HYDROLASE 24 | | -- | | | -- | | | -- | | | | | 1.3 |
|  |  | down | Bra017855 | | | | [AT4G37800.1](http://www.arabidopsis.org/servlets/TairObject?type=gene&id=131160) | | | XYLOGLUCAN ENDOTRANSGLUCOSYLASE/  HYDROLASE 7 | | -2.7 | | | -3.2 | | | -- | | | | | -- |
|  |  |  | Bra002718 | | | | [AT5G57560.1](http://www.arabidopsis.org/servlets/TairObject?type=gene&id=136049) | | | XYLOGLUCAN ENDOTRANSGLUCOSYLASE/  HYDROLASE 22 | | -- | | | -- | | | -2 | | | | | -- |
|  |  |  | Bra002719 | | | | [AT5G57560.1](http://www.arabidopsis.org/servlets/TairObject?type=gene&id=136049) | | | XYLOGLUCAN ENDOTRANSGLUCOSYLASE/  HYDROLASE 22 | | -- | | | -- | | | -2.9 | | | | | -- |
|  | |  | Bra017220 | | | | [AT2G36870.1](http://www.arabidopsis.org/servlets/TairObject?type=gene&id=35590) | | | XYLOGLUCAN ENDOTRANSGLUCOSYLASE/  HYDROLASE 32 | | -1.4 | | | -- | | | -- | | | | | -- |
|  | |  | Bra005238 | | | | [AT2G36870.1](http://www.arabidopsis.org/servlets/TairObject?type=gene&id=35590) | | | XYLOGLUCAN ENDOTRANSGLUCOSYLASE/  HYDROLASE 32 | | -1.8 | | | -1.2 | | | -- | | | | | -- |
| expansin | | up | Bra016767 | | | | [AT1G12560.1](http://www.arabidopsis.org/servlets/TairObject?type=gene&id=431224) | | | EXPANSIN A7 | | -- | | | -- | | | 1.45 | | | | | -- |
|  | |  | Bra019775 | | | | [AT1G12560.1](http://www.arabidopsis.org/servlets/TairObject?type=gene&id=431224) | | | EXPANSIN A7 | | -- | | | -- | | | 1.32 | | | | | -- |
|  | |  | Bra026965 | | | | [AT1G12560.1](http://www.arabidopsis.org/servlets/TairObject?type=gene&id=431224) | | | EXPANSIN A7 | | -- | | | -- | | | 1.9 | | | | | -- |
|  | |  | Bra027249 | | | | [AT3G15370.1](http://www.arabidopsis.org/servlets/TairObject?type=gene&id=39738) | | | EXPANSIN 12 | | -- | | | -- | | | -- | | | | | 1.15 |
|  | |  | Bra036638 | | | | [AT1G62980.1](http://www.arabidopsis.org/servlets/TairObject?type=gene&id=29374) | | | EXPANSIN 18 | | -- | | | -- | | | 1.38 | | | | | -- |
|  | | down | Bra012684 | | | | [AT4G17030.1](http://www.arabidopsis.org/servlets/TairObject?type=gene&id=130209) | | | EXPANSIN-LIKE B1 | | -1.2 | | | -1.2 | | | -- | | | | | -1.6 |
|  | |  | Bra026272 | | | | [AT4G28250.1](http://www.arabidopsis.org/servlets/TairObject?type=gene&id=129150) | | | EXPANSIN B3 | | -1.1 | | | -- | | | -- | | | | | -1.1 |
|  | |  | Bra024686 | | | | [AT1G26770.2](http://www.arabidopsis.org/servlets/TairObject?type=gene&id=1000648662) | | | EXPANSIN 10 | | -1.5 | | | -- | | | -- | | | | | -1.9 |
|  | |  | Bra016473 | | | | [AT1G20190.1](http://www.arabidopsis.org/servlets/TairObject?type=gene&id=138139) | | | EXPANSIN 11 | | -- | | | -- | | | -- | | | | | -2 |
|  | |  | Bra007170 | | | | [AT3G55500.1](http://www.arabidopsis.org/servlets/TairObject?type=gene&id=41434) | | | EXPANSIN 16 | | -1.7 | | | -1.9 | | | -- | | | | | -- |
|  | |  | Bra014739 | | | | [AT3G55500.1](http://www.arabidopsis.org/servlets/TairObject?type=gene&id=41434) | | | EXPANSIN 16 | | -2.2 | | | -- | | | -- | | | | | -- |
| fasciclin-like | | down | Bra028848 | | | | [AT5G03170.1](http://www.arabidopsis.org/servlets/TairObject?type=gene&id=131869) | | | FASCICLIN-LIKE ARABINOGALACTAN-PROTEIN 11 | | -1.1 | | | -1.1 | | | -- | | | | | -- |
|  | |  | Bra006656 | | | | [AT5G60490.1](http://www.arabidopsis.org/servlets/TairObject?type=gene&id=136150) | | | FASCICLIN-LIKE ARABINOGALACTAN-PROTEIN 12, | | -1.2 | | | -1 | | | -- | | | | | -- |
|  | |  | Bra005894 | | | | [AT5G06390.1](http://www.arabidopsis.org/servlets/TairObject?type=gene&id=134688) | | | FASCICLIN-LIKE ARABINOGALACTAN PROTEIN 17 PRECURSOR | | -- | | | -4.9 | | | -- | | | | | -- |
| arabinogalactan protein | | up | Bra028633 | | | | [AT5G64310.1](http://www.arabidopsis.org/servlets/TairObject?type=gene&id=135893) | | | ARABINOGALACTAN PROTEIN 1 | | 1.12 | | | -- | | | -- | | | | | -- |
|  |  |  | Bra038521 | | | | [AT2G22470.1](http://www.arabidopsis.org/servlets/TairObject?type=gene&id=33024) | | | ARABINOGALACTAN PROTEIN 2 | | 1.45 | | | -- | | | -- | | | | | -- |
|  | |  | Bra002808 | | | | [AT5G56540.1](http://www.arabidopsis.org/servlets/TairObject?type=gene&id=135039) | | | ARABINOGALACTAN PROTEIN 14 | | 1.03 | | | -- | | | -- | | | | | -- |
|  | | down | Bra008940 | | | | [AT5G11740.1](http://www.arabidopsis.org/servlets/TairObject?type=gene&id=137079) | | | ARABINOGALACTAN PROTEIN 15 | | -5.1 | | | -4.4 | | | -5.8 | | | | | -5.3 |
|  | |  | Bra023339 | | | | [AT5G11740.1](http://www.arabidopsis.org/servlets/TairObject?type=gene&id=137079) | | | ARABINOGALACTAN PROTEIN 15 | | -- | | | -1.3 | | | -1.3 | | | | | -- |
| cellulose synthase | | down | Bra006036 | | | | [AT5G09870.1](http://www.arabidopsis.org/servlets/TairObject?type=gene&id=136557) | | | CELLULOSE SYNTHASE 5 | | -1 | | | -- | | | -- | | | | | -- |
|  | |  | Bra008583 | | | | [AT5G16910.1](http://www.arabidopsis.org/servlets/TairObject?type=gene&id=132475) | | | CELLULOSE-SYNTHASE LIKE D2 | | -- | | | -- | | | -1.1 | | | | | -- |
|  |  |  | Bra037098 | | | | [AT1G55850.1](http://www.arabidopsis.org/servlets/TairObject?type=gene&id=28816) | | | CELLULOSE SYNTHASE LIKE E1, CSLE1 | | -1.9 | | | -2.1 | | | -1.4 | | | | | -- |
|  | |  | Bra006407 | | | | [AT5G17420.1](http://www.arabidopsis.org/servlets/TairObject?type=gene&id=136657) | | | CELLULOSE SYNTHASE CATALYTIC SUBUNIT 7, CESA7 | | -- | | | -1.1 | | | -- | | | | | -1 |
| pectin methylesterase | | up | Bra034553 | | | | [AT4G33220.1](http://www.arabidopsis.org/servlets/TairObject?type=gene&id=129490) | | | PECTIN METHYLESTERASE 44, PME44 | | -- | | | 1.19 | | | -- | | | | | -- |
|  |  | down | Bra021549 | | | | [AT3G14310.1](http://www.arabidopsis.org/servlets/TairObject?type=gene&id=39886) | | | PECTIN METHYLESTERASE 3, PME3 | | -1 | | | -- | | | -- | | | | | -- |
|  | |  | Bra000540 | | | | [AT2G26440.1](http://www.arabidopsis.org/servlets/TairObject?type=gene&id=36903) | | | PECTIN METHYLESTERASE 12, PME12 | | -- | | | -- | | | -- | | | | | -2.1 |
|  | |  | Bra003062 | | | | [AT5G53370.1](http://www.arabidopsis.org/servlets/TairObject?type=gene&id=133296) | | | PECTIN METHYLESTERASE PCR FRAGMENT | | -2.4 | | | -3.4 | | | -1.1 | | | | | -- |
| **Pathogenesis-related**  **(PR) proteins** | | | total of 18genes | | | | | | |  | |  | | |  | | |  | | | | |  |
| pathogenesis-related protein | up | | Bra015873 | | | | [AT3G57260.1](http://www.arabidopsis.org/servlets/TairObject?type=gene&id=38380) | | | PATHOGENESIS-RELATED PROTEIN 2, PR2 | | -- | | | -- | | | 1.46 | | | | | -- |
|  |  | | Bra007315 | | | | [AT3G57260.1](http://www.arabidopsis.org/servlets/TairObject?type=gene&id=38380) | | | PATHOGENESIS-RELATED PROTEIN 2, PR2 | | 3.21 | | | 2.3 | | | 1.83 | | | | | -- |
|  |  | | Bra001122 | | | | [AT3G04720.1](http://www.arabidopsis.org/servlets/TairObject?type=gene&id=38808) | | | PATHOGENESIS-RELATED 4, PR4 | | 1.96 | | | -- | | | 3.2 | | | | | -- |
|  |  | | Bra001123 | | | | [AT3G04720.1](https://www.arabidopsis.org/servlets/TairObject?type=gene&id=38808) | | | PATHOGENESIS-RELATED 4, PR4 | | -- | | | -- | | | 1.43 | | | | | -- |
|  |  | | Bra011464 | | | | [AT4G33720.1](https://www.arabidopsis.org/servlets/TairObject?type=gene&id=130710) | | | pathogenesis-related protein, putative | | 1.36 | | | -- | | | 1.12 | | | | | 2.56 |
|  |  | | Bra036981 | | | | [AT4G33720.1](http://www.arabidopsis.org/servlets/TairObject?type=gene&id=130710) | | | pathogenesis-related protein, putative | | 2.67 | | | -- | | | 1.14 | | | | | -- |
|  |  | | Bra036984 | | | | [AT4G33710.1](http://www.arabidopsis.org/servlets/TairObject?type=gene&id=130731) | | | pathogenesis-related protein, putative | | 1.14 | | | 1.92 | | | -- | | | | | 2.72 |
| thaumatin | up | | Bra013773 | | | | [AT4G24180.1](http://www.arabidopsis.org/servlets/TairObject?type=gene&id=432503) | | | THAUMATIN-LIKE PROTEIN 1 | | 1.07 | | | -- | | | -- | | | | | -- |
|  |  | | Bra015874 | | | | [AT1G75030.1](http://www.arabidopsis.org/servlets/TairObject?type=gene&id=30780) | | | THAUMATIN-LIKE PROTEIN 3 | | -- | | | -- | | | 4.71 | | | | | -- |
|  |  | | Bra010503 | | | | [AT4G36010.1](http://www.arabidopsis.org/servlets/TairObject?type=gene&id=130839) | | | pathogenesis-related thaumatin family protein | | -- | | | -- | | | -- | | | | | 1.24 |
|  |  | | Bra015659 | | | | [AT1G77700.1](http://www.arabidopsis.org/servlets/TairObject?type=gene&id=138817) | | | pathogenesis-related thaumatin family protein | | -- | | | -- | | | 1.3 | | | | | -- |
|  |  | | Bra025728 | | | | [AT1G19320.1](http://www.arabidopsis.org/servlets/TairObject?type=gene&id=29537) | | | pathogenesis-related thaumatin family protein | | 1.71 | | | 2.71 | | | 1.38 | | | | | -- |
| lipid transfer protein | up | | Bra005099 | | | | [AT2G38540.1](http://www.arabidopsis.org/servlets/TairObject?type=gene&id=36597) | | | LIPID TRANSFER PROTEIN 1, LTP1 | | 1.41 | | | 1.61 | | | -- | | | | | -- |
|  |  | | Bra017112 | | | | [AT2G38540.1](http://www.arabidopsis.org/servlets/TairObject?type=gene&id=36597) | | | LIPID TRANSFER PROTEIN 1, LTP1 | | 1.21 | | | 1.65 | | | -- | | | | | 1.07 |
|  |  | | Bra006721 | | | | [AT5G59320.1](http://www.arabidopsis.org/servlets/TairObject?type=gene&id=135217) | | | LIPID TRANSFER PROTEIN 3, LTP3 | | 1.25 | | | 1.83 | | | -- | | | | | -- |
|  |  | | Bra020322 | | | | [AT5G59310.1](http://www.arabidopsis.org/servlets/TairObject?type=gene&id=135214) | | | LIPID TRANSFER PROTEIN 4, LTP4 | | -- | | | 1.37 | | | -- | | | | | 1.07 |
|  |  | | Bra012847 | | | | [AT3G51600.1](http://www.arabidopsis.org/servlets/TairObject?type=gene&id=435382) | | | LIPID TRANSFER PROTEIN 5, LTP5 | | -- | | | 1 | | | -- | | | | | -- |
|  |  | | Bra001345 | | | | [AT3G08770.2](http://www.arabidopsis.org/servlets/TairObject?type=gene&id=1000688205) | | | LIPID TRANSFER PROTEIN 6, LTP6 | | -- | | | 2.26 | | | -- | | | | | 1.11 |
| **Chitinase** |  | | total of 10 genes | | | | | | |  | |  | | |  | | |  | | | | |  |
|  | up | | Bra039597 | | | | AT2G43590.1 | | | Chitinase family protein | | -- | | | -- | | | 5.07 | | | | | -- |
|  |  | | Bra007071 | | | | [AT3G54420.1](http://www.arabidopsis.org/servlets/TairObject?type=gene&id=435353) | | | CHITINASE CLASS IV, | | -- | | | 2 | | | -- | | | | | -- |
|  |  | | Bra013426 | | | | [AT4G19810.1](https://www.arabidopsis.org/servlets/TairObject?type=gene&id=130693) | | | CLASS V CHITINASE | | -- | | | 1.04 | | | -- | | | | | -- |
|  |  | | Bra000311 | | | | [AT2G43590.1](http://www.arabidopsis.org/servlets/TairObject?type=gene&id=434876) | | | Chitinase family protein | | 1.91 | | | 1.91 | | | 1.09 | | | | | -- |
|  | down | | Bra034754 | | | | [AT3G12500.1](http://www.arabidopsis.org/servlets/TairObject?type=gene&id=40157) | | | BASIC CHITINASE, PR3 | | -3.4 | | | -3.8 | | | -2.3 | | | | | -3.5 |
|  |  | | Bra038726 | | | | [AT3G12500.1](http://www.arabidopsis.org/servlets/TairObject?type=gene&id=40157) | | | BASIC CHITINASE, PR3 | | -1.2 | | | -- | | | -- | | | | | -- |
|  |  | | Bra001453 | | | | [AT3G12500.1](http://www.arabidopsis.org/servlets/TairObject?type=gene&id=40157) | | | BASIC CHITINASE, PR3 | | -2.1 | | | -3 | | | -- | | | | | -- |
|  |  | | Bra000310 | | | | [AT2G43570.1](http://www.arabidopsis.org/servlets/TairObject?type=gene&id=434702) | | | CHITINASE, PUTATIVE | | -- | | | -- | | | -- | | | | | -2.3 |
|  |  | | Bra005345 | | | | [AT2G43590.1](http://www.arabidopsis.org/servlets/TairObject?type=gene&id=434876) | | | Chitinase family protein | | -- | | | -3.7 | | | -- | | | | | -- |
|  | up/down | | Bra004771 | | | | AT2G43570.1 | | | CHITINASE, PUTATIVE | | -- | | | -- | | | 1.14 | | | | | -1 |
