# Supplementary material for: Transcriptome Analysis of Brassica rapa Near-Isogenic Lines Carrying Clubroot-Resistant and –Susceptible Alleles in Response to Plasmodiophora brassicae during Early Infection
Source: Front Plant Sci. 2016 Jan 5;6:1183. doi: 10.3389/fpls.2015.01183 (PMC4700149; doi:10.3389/fpls.2015.01183)
Supplement: Figure S1 — Disease symptoms in CR BJN3-2 and BJN3-2 30 days after P. brassicae inoculation. There were no visible clubs on the CR BJN3-2 (left), but severe clubbing occurred on the main roots and lateral roots of BJN3-2 (right). [file Presentation1.zip › Supplementary Material/Supplementary Table S8.docx]

***Supplementary Materials***

**Transcriptome analysis of *Brassica rapa* near-isogenic lines carrying clubroot-resistant and –susceptible alleles in response to *Plasmodiophora brassicae* during early infection**

**Jingjing Chen^1†^, Wenxing Pang^1†^, Bing Chen^2^, Chunyu Zhang^3*^ and Zhongyun Piao^1*^**

^†^Jingjing Chen and Wenxing Pang contributed equally to this work

*** Correspondence:**

Zhongyun Piao: zypiao@syau.edu.cn

Chunyu Zhang: zhchy@mail.hzau.edu.cn

**Supplementary Table S8:** Expression levels of fifteen candidate clubroot resistance genes at *CRb* locus.

| **Gene Names** | Susceptible (S) NIL | | | |  | Resistant (R) NIL | | | |
| --- | --- | --- | --- | --- | --- | --- | --- | --- | --- |
|  | 0 hai* | 12 hai | 72 hai | 96 hai |  | 0 hai | 12 hai | 72 hai | 96 hai |
| Bra012532 | 27.41 | 23.29 | 23.33 | 22.13 |  | 16.31 | 15.65 | 19.22 | 14.99 |
| Bra012533 | 0.34 | 0.08 | 0.19 | 0.00 |  | 0.37 | 0.66 | 0.71 | 0.20 |
| Bra012534 | 2.27 | 3.28 | 2.76 | 0.19 |  | 3.86 | 6.75 | 0.75 | 0.06 |
| Bra012535 | 0.00 | 0.09 | 0.10 | 0.00 |  | 0.00 | 0.98 | 0.29 | 0.00 |
| Bra012536 | 2.52 | 3.74 | 2.76 | 2.98 |  | 1.04 | 1.82 | 1.50 | 1.93 |
| Bra012537 | 0.00 | 0.00 | 0.00 | 0.00 |  | 0.00 | 0.00 | 0.00 | 0.00 |
| Bra012538 | 0.00 | 0.13 | 0.16 | 0.00 |  | 0.00 | 0.00 | 0.15 | 0.00 |
| Bra012539 | 12.13 | 11.58 | 10.74 | 13.09 |  | 10.03 | 11.75 | 11.33 | 11.89 |
| Bra012540 | 0.00 | 0.11 | 0.00 | 0.06 |  | 0.11 | 0.14 | 0.10 | 0.09 |
| Bra012541 | 6.55 | 5.21 | 5.63 | 3.55 |  | 8.90 | 8.05 | 8.32 | 6.46 |
| Bra012542 | 0.28 | 0.54 | 0.71 | 0.46 |  | 0.41 | 0.75 | 0.90 | 0.26 |
| Bra012543 | 25.97 | 22.50 | 23.78 | 12.33 |  | 36.29 | 30.34 | 24.32 | 11.12 |
| Bra012544 | 0.13 | 0.04 | 0.86 | 0.08 |  | 0.43 | 0.26 | 0.33 | 0.17 |
| Bra012545 | 0.00 | 0.00 | 0.00 | 0.00 |  | 0.00 | 0.00 | 0.00 | 0.00 |
| Bra012546 | 0.00 | 0.00 | 0.00 | 0.00 |  | 0.00 | 0.00 | 0.00 | 0.00 |

*** hours after inoculation**
