# Supplementary figures and images for: Transcriptome Analysis of Brassica rapa Near-Isogenic Lines Carrying Clubroot-Resistant and –Susceptible Alleles in Response to Plasmodiophora brassicae during Early Infection
Source: Front Plant Sci. 2016 Jan 5;6:1183. doi: 10.3389/fpls.2015.01183 (PMC4700149; doi:10.3389/fpls.2015.01183)

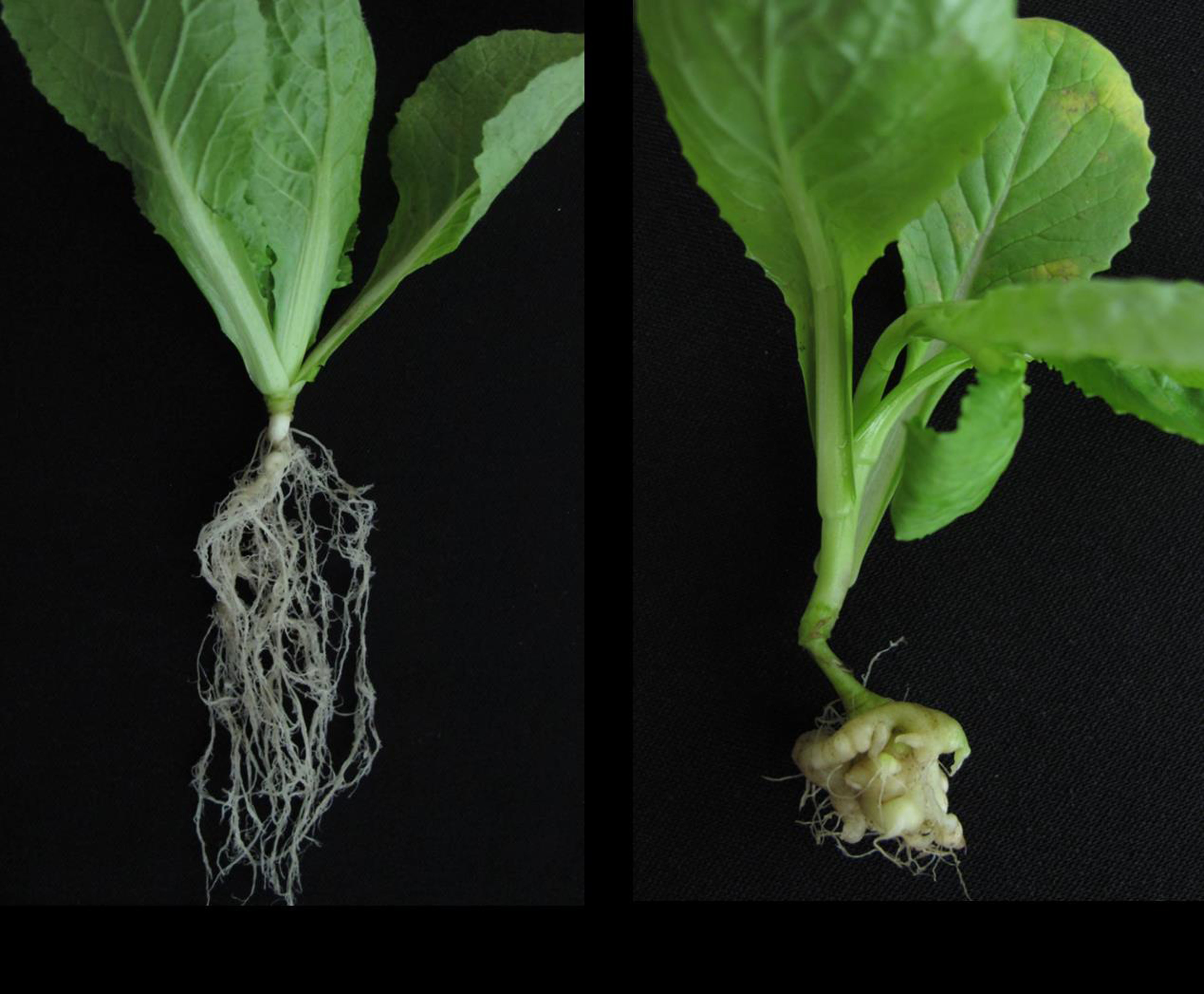

Supplement: Figure S1 — Disease symptoms in CR BJN3-2 and BJN3-2 30 days after P. brassicae inoculation. There were no visible clubs on the CR BJN3-2 (left), but severe clubbing occurred on the main roots and lateral roots of BJN3-2 (right). [file Presentation1.zip › Supplementary Material/Supplementary Figure S1.jpg]

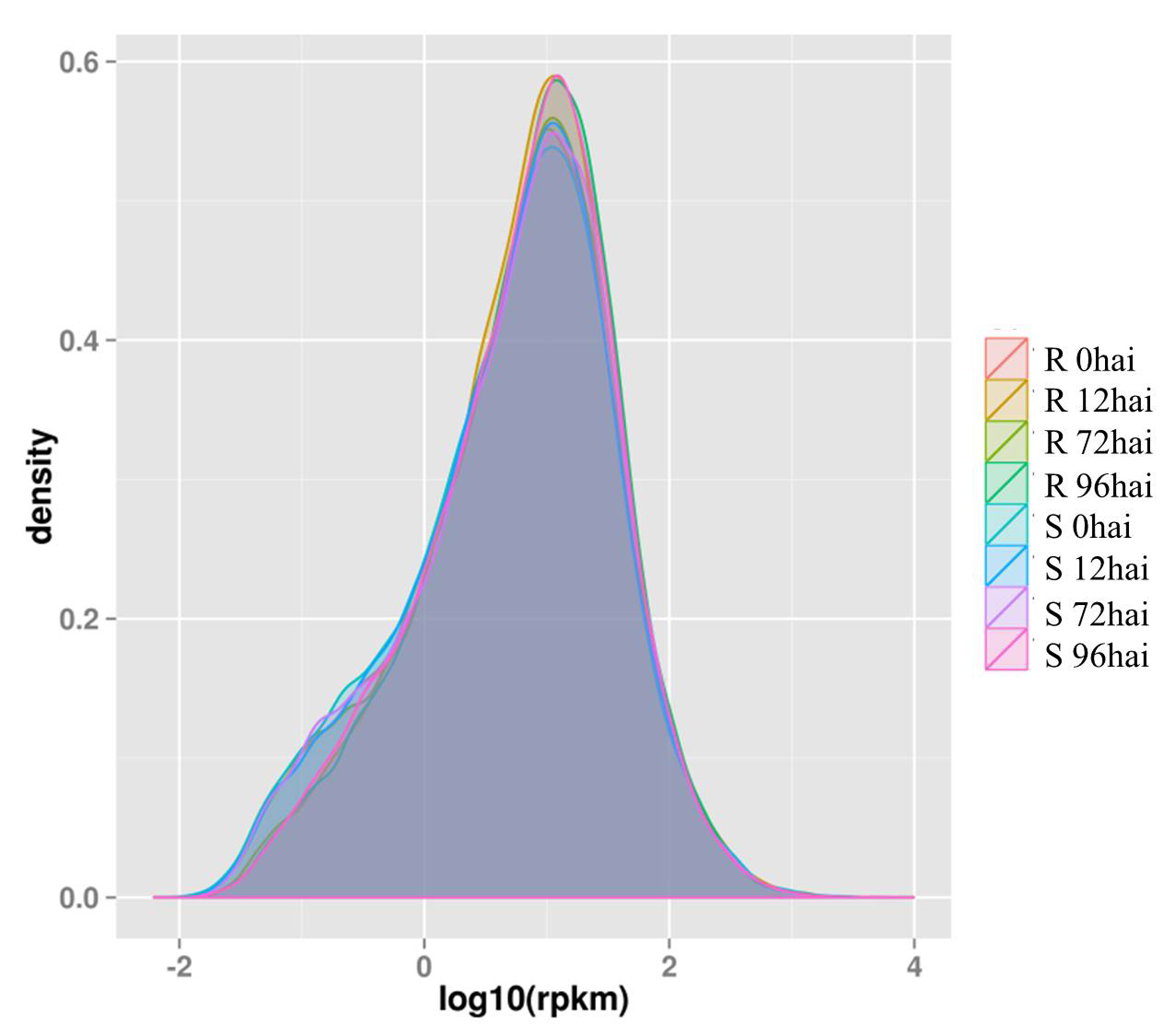

Supplement: Figure S1 — Disease symptoms in CR BJN3-2 and BJN3-2 30 days after P. brassicae inoculation. There were no visible clubs on the CR BJN3-2 (left), but severe clubbing occurred on the main roots and lateral roots of BJN3-2 (right). [file Presentation1.zip › Supplementary Material/Supplementary Figure S2.jpg]

Supplementary Figure S4: The top GO directed acyclic graph of DEGs at 0 hai in biological process

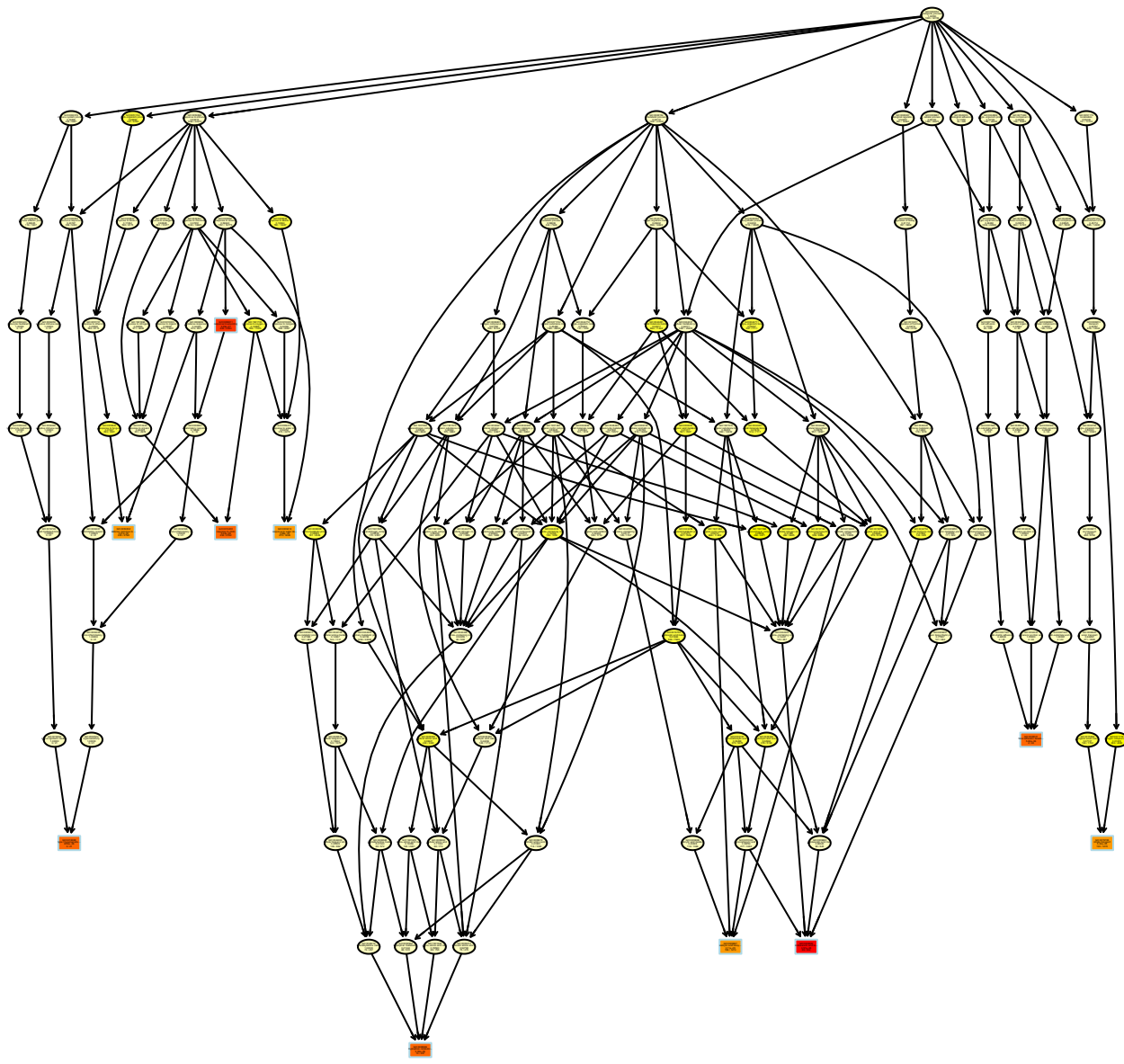

Supplement: Figure S1 — Disease symptoms in CR BJN3-2 and BJN3-2 30 days after P. brassicae inoculation. There were no visible clubs on the CR BJN3-2 (left), but severe clubbing occurred on the main roots and lateral roots of BJN3-2 (right). [file Presentation1.zip › Supplementary Material/Supplementary Figure S4.pdf]

Supplementary Figure S5: The top GO directed acyclic graph of DEGs at 12 hai in biological process

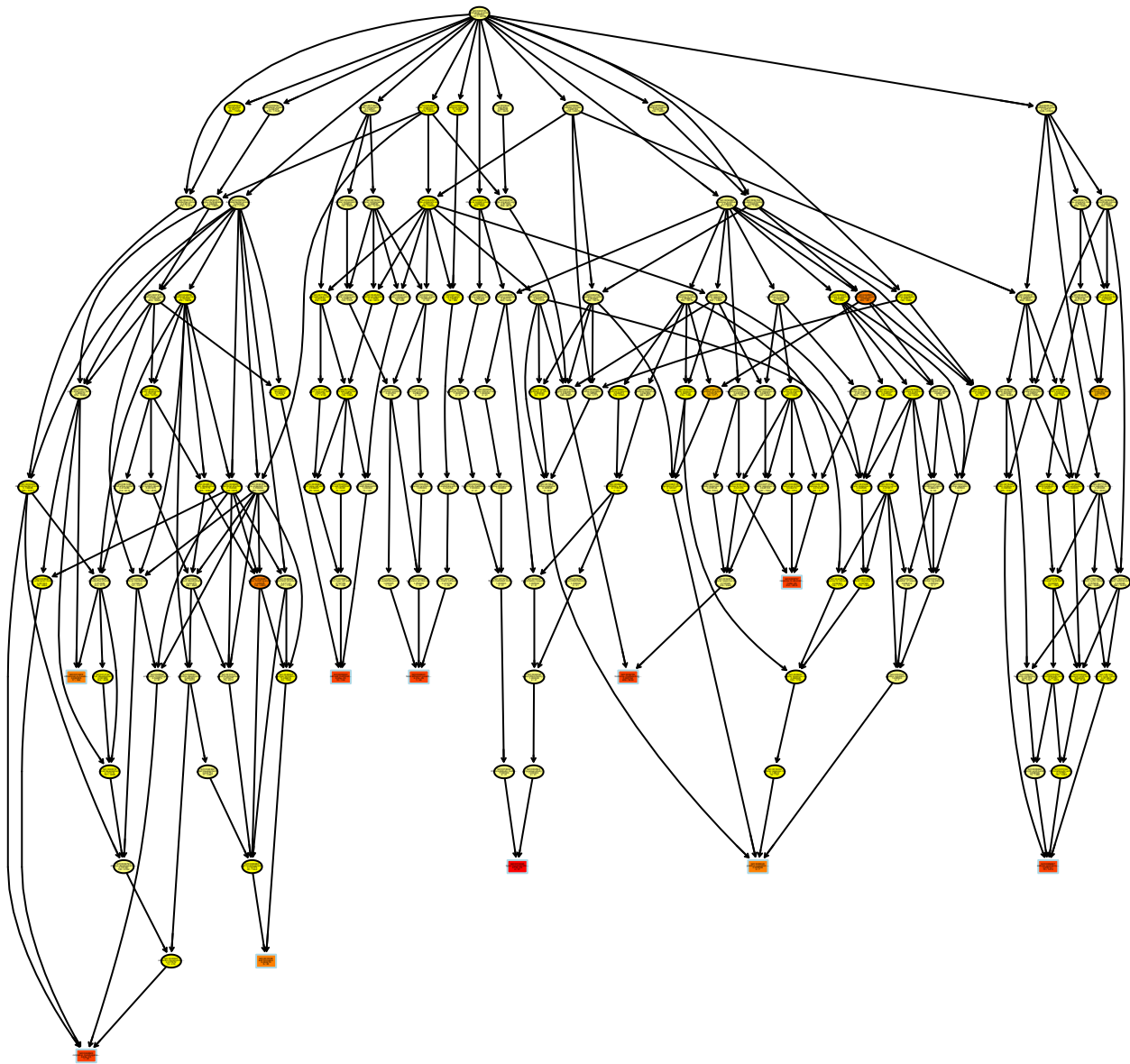

Supplement: Figure S1 — Disease symptoms in CR BJN3-2 and BJN3-2 30 days after P. brassicae inoculation. There were no visible clubs on the CR BJN3-2 (left), but severe clubbing occurred on the main roots and lateral roots of BJN3-2 (right). [file Presentation1.zip › Supplementary Material/Supplementary Figure S5.pdf]

Supplementary Figure S6: The top GO directed acyclic graph of DEGs at 72 hai in biological process

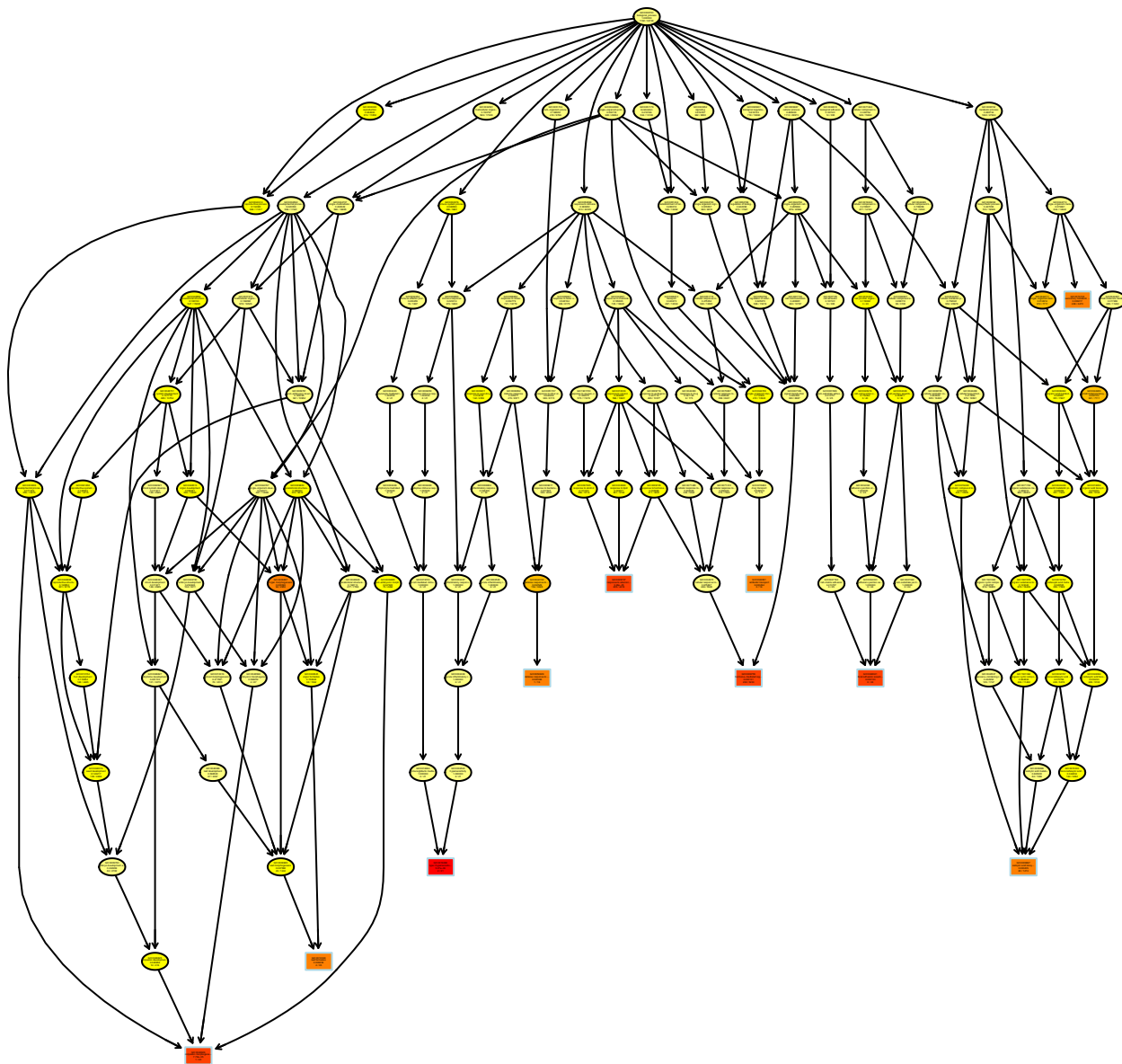

Supplement: Figure S1 — Disease symptoms in CR BJN3-2 and BJN3-2 30 days after P. brassicae inoculation. There were no visible clubs on the CR BJN3-2 (left), but severe clubbing occurred on the main roots and lateral roots of BJN3-2 (right). [file Presentation1.zip › Supplementary Material/Supplementary Figure S6.pdf]

Supplementary Figure S7: The top GO directed acyclic graph of DEGs at 96 hai in biological process

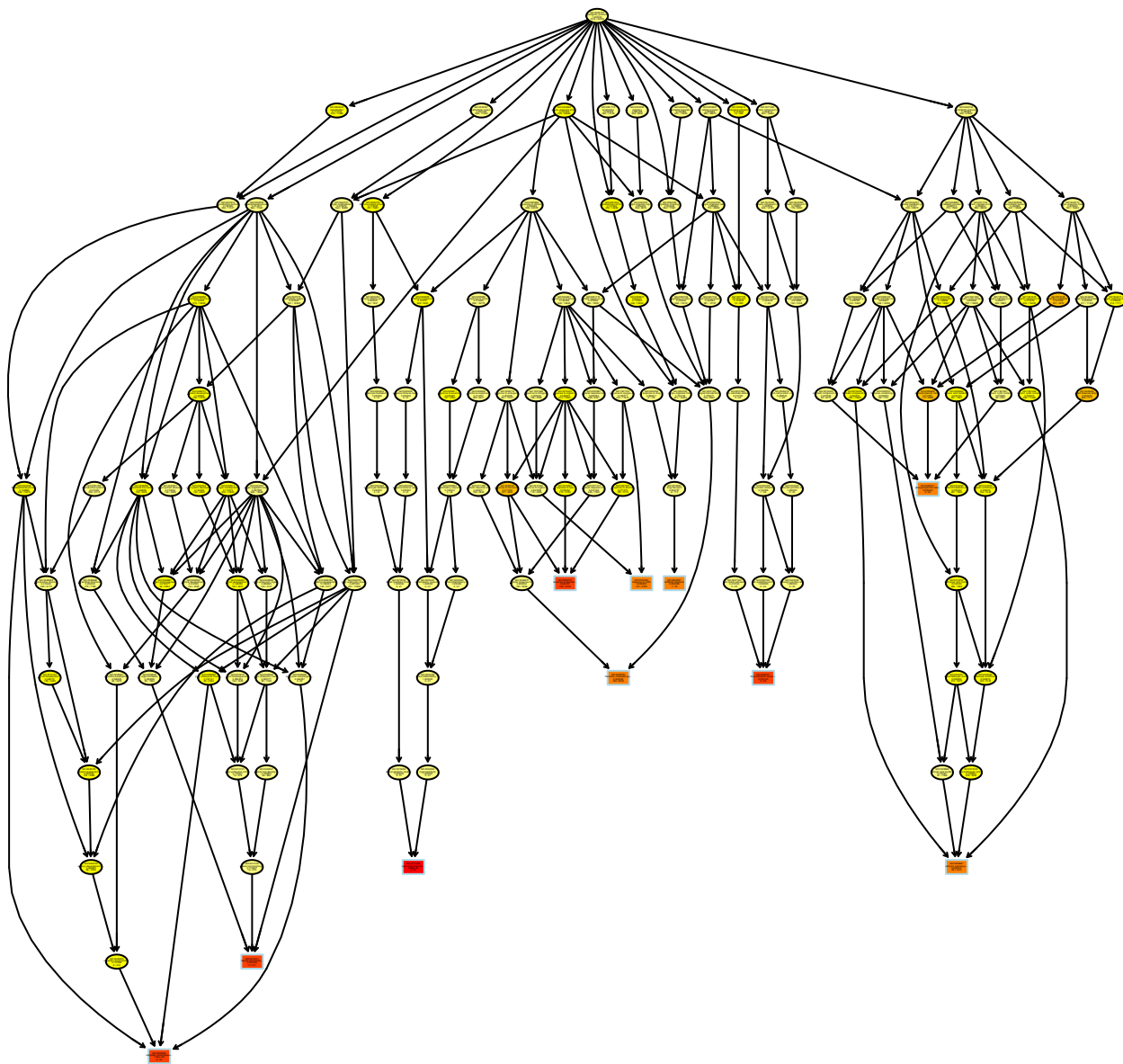

Supplement: Figure S1 — Disease symptoms in CR BJN3-2 and BJN3-2 30 days after P. brassicae inoculation. There were no visible clubs on the CR BJN3-2 (left), but severe clubbing occurred on the main roots and lateral roots of BJN3-2 (right). [file Presentation1.zip › Supplementary Material/Supplementary Figure S7.pdf]
